# Triplex real-time qPCR for the simultaneous detection of *Botryosphaeriaceae* species in woody crops and environmental samples

**DOI:** 10.3389/fpls.2024.1435462

**Published:** 2024-10-11

**Authors:** Laura Romero-Cuadrado, Ana Aguado, David Ruano-Rosa, Nieves Capote

**Affiliations:** Department of Sustainable Crop Protection, Andalusian Institute of Agricultural and Fisheries Research and Training (IFAPA), Seville, Spain

**Keywords:** Botryosphaeria dieback, preventive control, multiplex qPCR, woody crops, nursery, soil, rainwater, trapped spores

## Abstract

**Introduction:**

Species of *Botryosphaeriaceae* fungi are relevant pathogens of almond causing trunk cankers, extensive gumming, necrosis of internal tissues and plant dieback and dead, threatening almond productivity. A novel triplex quantitative real-time PCR (qPCR) assay was designed for the simultaneous detection and quantification of *Neofusicoccum parvum*, *Botryosphaeria dothidea* and the *Botryosphaeriaceae* family.

**Material and methods:**

The method was validated in symptomatic and asymptomatic almond, avocado, blueberry and grapevine plants and in environmental samples, such as cropping soil and rainwater and in artificially inoculated trapped spores, demonstrating the same performance on several matrices.

**Results and discussion:**

The limit of detection of the triplex qPCR was 10 fg of genomic DNA for the three fungal targets, with high correlation coefficients (R2) and amplification efficiencies between 90 and 120%. Although the triplex qPCR demonstrated to be more sensitive and accurate than the traditional plate culturing and further sequencing method, a substantial agreement (kappa index = 0.8052 ± 0.0512) was found between the two detection methods. The highly sensitive qPCR assay allows for accurate diagnosis of symptomatic plants and early detection of *Botryosphaeriaceae* fungi in asymptomatic plants (rootstocks and grafting scions from almond nurseries). Furthermore, the triplex qPCR successfully detected *Botryosphaeriaceae* fungi in environmental samples, such as cropping soils and rainwater. It was also capable of detecting as few as 10 conidia in artificially inoculated tapes. Therefore, the triplex qPCR is a valuable tool for accurate diagnosis, aiding in the implementation of suitable control measures. It enables preventive detection in asymptomatic samples, helping to avoid the introduction and spread of these pathogens in production fields. Moreover, it assists in identifying inoculum sources and quantifying inoculum levels in crop environments, contributing to a precise phytosanitary application schedule, thereby reducing production costs and preserving the environment.

## 1 Introduction

Fungal diseases can result in significant losses in the yield and quality of crop and forest trees, additionally reducing the lifespan of plants. Depending on their incidence and severity, these diseases can become limiting factors for agricultural production (Aiello et al., 2023; Carlucci et al., 2015; Guarnaccia et al., 2022; Gusella et al., 2020; Linaldeddu et al., 2016; Vettraino et al., 2017; Zhang et al., 2023). The current and impending climate change situation exacerbates this issue, leading to a progressive increase in fungal diseases in recent decades (Fernández et al., 2023; Moral et al., 2019; Velásquez et al., 2018).

Almond [*Prunus dulcis* (Mill.) D.A. Webb] cultivation in Spain has significantly increased over the last decade, making it a highly profitable fruit crop. The intensification of the crop has positioned Spain as the third-largest producer of almond in the word, following the United States and Australia. However, the adoption of new management techniques, such as drip irrigation and fertilization, the use of more productive varieties and the implementation of high-density production systems, coupled with the ongoing climatic change and the EU regulations prohibiting the use of many chemical fungicides, has increased the incidence of fungal diseases in this crop (Doll et al., 2013; Velásquez et al., 2018). Among them, Botryosphaeria dieback is a fungal disease that is increasingly threatening woody crops worldwide. *Botryosphaeria dothidea* was firstly described as the causal agent of “band canker” of almond, causing horizontal cankers expanding in the almond trunk (English, 1975). However, several fungal species of the *Botryosphaeriaceae* family are involved in the etiology of the disease.

The *Botryosphaeriaeceae* family comprises filamentous fungi widely distributed worldwide, which can function as saprophytes, endophytes or phytopathogens (Slippers and Wingfield, 2007). These fungi have a wide host range, mainly affecting woody plant species, although they can also infect herbaceous plants and even lichens. This family is made up of about 24 genera, with *Diplodia*, *Dothiorella*, *Lasiodiplodia* and *Neofusicoccum* identified as having the most extensive host range (Batista et al., 2021)*. Botryosphaeriaceae* species are mostly aerial, although there are some soil-borne species, such as *Macrophomina phaseolina*. Infection of the host plant occurs through natural or pruning wounds as well as through natural openings, such as lenticels and stomata. They produce latent infections that become pathogenic when the plant is under stress, both abiotic (such as drought or nutritional deficiency) or biotic (e.g., the infection by another pathogen). In fact, drought or heat stress negatively affects plant physiology, improving pathogen colonization and increasing host susceptibility (Agustí-Brisach et al., 2020a; Batista et al., 2021; Fernández et al., 2023; Haidar et al., 2020). This silent infection makes their detection more difficult, especially in asymptomatic nursery stocks or young trees in the field.

*Botryosphaeriaceae* pathogenic fungi secrete hydrolytic enzymes that degrade the plant cell wall and colonize the xylem vessels, reducing the flow of nutrients to the plant. Additionally, these fungi can produce phytotoxic metabolites that facilitate invasion and colonization of the host plant, increasing their virulence (Belair et al., 2023; Mesguida et al., 2023; Reveglia et al., 2022). The infected wood is the primary source of inoculum, where specialized structures or fruiting bodies called pycnidia (producing conidia, the asexual spores) and perithecia (that produce ascospores, the sexual spores) develop. The fruiting bodies can persist either on the wood in the tree or on pruning residues on the ground (Marsberg et al., 2017). Also, the soil serves the main inoculum source of soil-borne species where they can persist as resistant structures for extended periods (Márquez et al., 2021). The main sources of external inoculum of *Botryosphaeriaceae* are susceptible neighboring crops and asymptomatic nursery stocks (Billones-Baaijens et al., 2013c; Carbone et al., 2022; Luo et al., 2024; Pintos et al., 2018; Tennakoon et al., 2018). Once in the field, these fungi are transmitted through the air, rainwater, irrigation water, insect vectors, and pruning or harvesting tools (Agustí-Brisach et al., 2015; Baskarathevan et al., 2013; Úrbez-Torres et al., 2010). It is also important to highlight the worldwide spread through the international movement of plants without adequate quarantine systems (Aiello et al., 2023).

Symptoms typically manifest in spring and early summer, associated with a temperature increase, and consist of cankers on the trunk and branches (necrotic lesion in the wood), abundant gummosis, necrosis of internal tissues (sectorized necrosis, pitting, subcortical rot), decay and occasional death of the plant, especially in young individuals (Avenot et al., 2022). The co-occurrence of different *Botryosphaeriaceae* species is common in canker diseased forest and woody crops (Fiorenza et al., 2023; Linaldeddu et al., 2023; Moral et al., 2019; Smahi et al., 2017; Úrbez-Torres, 2011). Among them, *Botryosphaeria dothidea* has been identified as the predominant species causing Botryosphaeria dieback in almond crop in Spain (Agustí-Brisach et al., 2020a). On the other hand, *Neofusicoccum* spp. has emerged as the most aggressive species, not only in almond but also in other woody crops (Aiello et al., 2022; Arjona-Girona et al., 2019; Billones-Baaijens et al., 2013a; Espinoza et al., 2009; Olmo et al., 2016; Úrbez-Torres et al., 2016; Valencia et al., 2019).

The control of fungal diseases mainly relies on the application of chemical fungicides, significantly increasing production costs. Furthermore, the progressive restriction in the manufacture and use of many active compounds, imposed by European regulations due to the harm caused to human health, animals, and the environment, have intensified the search for sustainable control alternatives. Biological control, consider as a promising sustainable option (Romero-Cuadrado et al., 2023), requires extensive *in vitro* and *in vivo* tests to demonstrate its efficacy under field conditions. In addition, no studies on host resistance of cultivars are current available. Therefore, preventive control, particularly early detection of the causal agent, proves highly effective in preventing the introduction of the pathogen into a production field and its subsequent establishment and spread. Traditional detection methods based on *in vitro* tissue culture are laborious and not always accurate. The use of molecular techniques, such as real-time PCR (qPCR), for the specific and highly sensitive detection of fungal pathogens in plant material and environmental samples is crucial for disease diagnosis and preventive control. Furthermore, given the morphological similarities among *Botryosphaeriaceae* species and the indistinguishable disease symptoms caused by these fungi and other woody pathogens, accurate identification of *Botryosphaeriaceae* species is essential. In addition, qPCR enables the quantification of the target, providing insights into the inoculum density. Quantifying inoculum density is decisive in epidemiological studies to implement timely and cost-effective fungicidal measures, preventing unnecessary expenses in cultivation and minimizing environmental impact. For these reasons, we developed a triplex qPCR protocol, based on our previously reported primers and hydrolysis probes (Romero-Cuadrado et al., 2023), for the simultaneous detection of *B. dothidea, N. parvum* (the most predominant and the most aggressive species in almond crop in Spain, respectively) and any species within the *Botryosphaeriaceae* family in the same reaction. The dynamic range (efficiency and coefficient of determination R^2^), repeatability, sensitivity and specificity of the triplex qPCR were verified. The ability to detect a fungal target present at lower abundance compared to other more abundant targets, potentially occurring in plants infected by more than one pathogen, was also assessed. Additionally, considering the potential impact of these fungi on other economically important crops in Spain, the qPCR protocol was validated for the detection of *Botryosphaeriaceae* fungi in avocado, blueberry and grapevine plants. Moreover, it has been validated in environmental samples which might constitute a source of inoculum of these fungi, such as soil, rainwater, and air.

## 2 Material and methods

### 2.1 Fungal isolates

A total of 34 *Botryosphaeriaceae* fungal isolates from almond, 60 from other woody host species and 23 from other genera commonly recovered in woody crops and obtained from different fungal collections were used in this study (**Supplementary Table S1**). The identification of fungal isolates was performed in both this and a previous study (Romero-Cuadrado et al., 2023) through sequencing the internal transcribed spacer (ITS1 and ITS2) and the 5.8S gene of the nuclear rDNA using ITS1 and ITS4 primers (White et al., 1990) and a portion of the translation elongation factor 1 alpha gene (*tef1*) using EF446f and EF1035r primers (Inderbitzin et al., 2005). Sequences were compared with those available at GenBank database by BLAST analysis (https://blast.ncbi.nlm.nih.gov/Blast.cgi). For fungal DNA extraction, a portion of the fungal mycelium was scrapped from a 7-day-old colony grown in PDA and placed in a centrifuge tube containing 20 µL of 25 mM NaOH, pH 12. The tubes were incubated at 100°C for 10 min, followed by a 4°C incubation for 5 min. Subsequently, 40 µL of 40 mM Tris-HCl at pH 5 were added, and 5 µL of this extract were used for PCR amplification.

### 2.2 Plant samples and DNA extraction

Almond samples consisted of trunk pieces from 26 symptomatic and 6 asymptomatic almond trees collected from four Spanish commercial orchards in La Rinconada and Alcalá del Río (Sevilla province), Jerez de la Frontera (Cádiz province) and Palma del Río (Córdoba province). Three symptomatic almond trees from Palma del Río orchard were uprooted and their roots and the soil below them were also analyzed. In addition, almond nursery samples were collected in October 2022 and June 2023 from the same nursery. Samples in the first date consisted of 30 asymptomatic grafting scions from ‘Soleta’, ‘Belona’, ‘Vairo’, ‘Marinada’, ‘Guara’ and ‘Lauranne’ mother plants (5 grafting scions per variety). Samples collected in June 2023 consisted of 30 asymptomatic grafting scions from ‘Soleta’, ‘Guara’ and ‘Lauranne’ mother plants (10 grafting scions per variety), as well as the main root, the secondary root and the trunk of ten GxN-15 almond rootstocks grown in the field soil previous to grafting. Avocado samples comprised 120 asymptomatic scions from asymptomatic trees of the ‘Hass’ and ‘Reed’ varieties collected in October 2022, and 100 asymptomatic scions collected from affected and non-affected trees of the ‘Hass’ variety collected in October 2023, making a total of 220 avocado scions analyzed. The avocado production field was located in Algarrobo, Málaga, Spain. Blueberry samples consisted of stems and roots from 12 symptomatic plants of the ‘Cupla’, ‘Manila’, ‘Ventura’ ‘Miss Alice’ ‘Olympus’ and ‘Emerald’ varieties, as well as the trunk of one asymptomatic plant collected from four commercial orchards located in Moguer and Gibraleón (Huelva, Spain). All blueberry plants were uprooted for analysis. Grapevine samples included 10 symptomatic and two asymptomatic shoots collected from the ‘Verdejo’ and ‘Tempranillo’ varieties from commercial and private orchards in Jerez de la Frontera, Trujillo and Valladolid Spanish localities.

DNA from plant samples was extracted following the method outlined by Romero-Cuadrado et al., 2023. Briefly, trunk subcortical tissue or grafting spikes were introduced into Bioreba plastic bags (Bioreba, Reinach, Switzerland) and ground in the presence of an extraction buffer composed of 1:20 (w:v) PBS buffer, pH 7.2 supplemented with 2% (w:v) polyvinyl pyrrolidone and 0.2% (w:v) sodium diethyl dithiocarbamate. The plant crude extract was kept on ice, and 400 μL were immediately used for DNA extraction according to the manufacturer’s instructions for the DNeasy Plant Pro kit (Qiagen, Hilden, Germany).

### 2.3 *In vitro* culture of plant tissues

Briefly, samples from trunk subcortical tissue or grafting spikes were divided into two halves: one half was processed for DNA extraction and triplex qPCR amplification as explained below. The other one was surface sterilized in 1.5% sodium hypochlorite for 2 min, rinsed twice in sterile distilled water and air-dried in a flow cabinet. Small pieces (0.5 cm) were placed on PDA plates and incubated at 25 ◦C for 7–10 days in darkness. Colonies resembling *Botryosphaeriaceae* species were subcultured in PDA, hyphal-tipped and sequenced for identification as explained above.

### 2.4 Agreement between the two detection methods

The agreement between triplex qPCR and plate culturing detection techniques was measured by calculating Cohen’s kappa (k) index (Landis and Koch, 1977) categorized as slight (k = 0.00-0.20), fair (k = 0.21-0.40), moderate (k = 0.41-0.60), substantial (k = 0.61-0.80), and almost perfect (k = 0.81-1.00) agreement.

### 2.5 Environmental samples and DNA extraction

Soil samples were collected from two almond, one avocado, one blueberry and three grapevine orchards underneath diseased plants (3 to 5 soil samples/crop field). If the plants were uprooted, such as in three almond and all blueberry plants, soil was sampled near the roots. For plants that were not uprooted, like the remaining almond, avocado and grapevine plants, soil was taken from the top 5 cm of depth with the help of a soil shovel. Soil samples were homogenized and subsequently lyophilized in a Telstar Lyoquest freeze dryer previously to DNA extraction.

DNA was extracted from 250-mg soil aliquots (3 replicates per sample), using the DNeasy PowerSoil Pro Kit (Qiagen, Hilden, Germany), following the manufacturer’s instructions using a Disruptor Genie^®^ homogenizer (Scientific industries)

Rainwater samples were collected in November and December 2023 and January and February 2024 in a commercial almond orchard (La Rinconada, Sevilla) known to contain infected trees. The rainwater was collected in 1-liter capacity bottles with a funnel of 15 cm in diameter attached to the opening, tied to each almond tree with plastic ties. The water was centrifuged in 50 ml centrifuge tubes at 8,000 rpm for 15 minutes. The pellets were resuspended in 1 ml of remaining water, homogenized using a vortex, and transferred to Eppendorf tubes for further centrifugation at 14,000 rpm for 5 minutes. The water was carefully removed using a pipette, and the pellet was preserved at -20°C until DNA extraction.

Three commercial DNA extraction kits were evaluated for efficiency: DNeasy PowerWater kit (Qiagen, Hilden, Germany), DNeasy Plant Pro Kit (Qiagen, Hilden, Germany) and DNeasy PowerSoil Pro Kit (Qiagen, Hilden, Germany). The manufacturer’s instructions were followed incorporating an additional step of incubation with 5 μL of RNase A (10 mg/ml) and 20 μL of proteinase K (20 mg/ml) at 65°C for 10 minutes, preceding homogenization in a Disruptor Genie^®^, (Scientific industries).

To assess the efficacy of detecting *Botryosphaeriaceae* trapped-spores in spore tapes, serial dilutions of conidia from *N. parvum* isolate NpALM2 were artificially inoculated onto glass microscope slides covered with Melinex tapes (2 x 3,5 cm, Burkard Scientific, U.K.) coated with silicone oil (Labkem). For conidia production, the protocol described by Yang et al., 2017 was followed with some modifications. Briefly, the *N. parvum* NpALM2 isolate was cultivated on 90 mm diameter PDA Petri plates and incubated for 3 to 4 days at room temperature until the mycelium covered the plate. The mycelium was disrupted using sterilized cotton swabs. The plates were then rinsed with sterile distilled water and allowed to dry in a laminar flow cabinet for 1 hour. The plates were covered with pre-sterilized filter paper and further incubated until sporogenesis was observed, monitored at 2-3 day intervals. Pycnidia were then collected using tweezers, transferred to tubes containing 5 mL of sterile distilled water, and ruptured using a pistil. The spore concentration was determined using a hemocytometer (Thoma Chamber).

Conidia suspension was adjusted at 10^6^ conidia/mL and serial 10-fold dilutions were obtained (10^6^ to 10^1^ con/mL). 100 μL of these suspensions were loaded onto Melinex tapes coated with silicone and air dried. For DNA extraction, two commercial kits were compared for efficiency: DNeasy PowerSoil Pro Kit (Qiagen, Hilden, Germany) and DNeasy Plant Pro kit (Qiagen, Hilden, Germany) following the manufacturer’s instructions.

DNA concentration from any source was fluorometrically quantified using a Qubit 4 fluorometer (Invitrogen, Thermo Fisher Scientific, Waltham, MA, USA). Five µL DNA extracted from plant, soil, water or trapped spores were used as samples for the triplex qPCR reactions.

### 2.6 Primers and hydrolysis probes

Primers and probes for the triplex qPCR were previously designed (Romero-Cuadrado et al., 2023), selected to have similar melting temperatures (Tm) to enable their use in multiplex reactions under the same PCR conditions, allowing for the simultaneous amplification of several targets. Hydrolysis probes specific for the detection of *B. dothidea*, *N. parvum* and *Botryosphaeriaceae* family were labeled with FAM, HEX and Cy5 fluorophores, respectively. FAM and HEX hydrolysis probes harbored an internal ZEN quencher and an Iowa Black FQ quencher (IBFQ) at the 3′-end. Cy5 hydrolysis probe carried a TAO internal quencher and an Iowa Black RQ 3’-end quencher (IBRQ) (**Supplementary Table S2**).

### 2.7 Multiplex qPCR optimization

Various concentrations of probes (200 nM and 250 nM) and primers (300 nM, 600 nM and 900 nM) were tested. Additionally, two different commercial master mixes, iTaq Universal Probes Supermix (Bio-Rad) and iQ Supermix (Bio-Rad) were assessed to optimize efficiency and enhance sensitivity. qPCR assays were performed in 96-well plates using a CFX OPUS 96 cycler (Bio-Rad, Hercules, CA, USA) in a final reaction volume of 20 μL. Reaction cocktails contained 5 μL of extracted DNA (from a pure fungal colony, plant tissue, soil, water or trapped spore samples), iQ Multiplex Powermix (1×) (Bio-Rad, Hercules, CA, USA), forward and reverse primers for *N. parvum*, *B. dothidea* and *Botryosphaeriaceae* family (300 nM, 600 nM or 900 nM), hydrolysis probes for *N. parvum*, *B. dothidea* and *Botryosphaeriaceae* family (200 nM or 250 nM) (**Supplementary Table S2**). Amplifications were performed at 95°C for 2 min, then 45 cycles of 10 s at 95°C, followed by 40 s at 60°C. Data collection was enabled at the extension step. In each run, sterile nuclease-free water was used as a non-template control (NTC). Data were analyzed using CFX Maestro 2.2 software (Bio-Rad, Hercules, California, USA).

### 2.8 Multiplex qPCR validation

For the validation of the triplex qPCR, efficiency and coefficient of determination (R^2^), limit of detection (LOD), asymmetric limit of detection (LODasym) and analytical specificity (inclusivity/exclusivity) were assessed following the criteria of The MIQE Guidelines (Bustin et al., 2009). In addition, the applicability of the technique was also proposed.

#### 2.8.1 Efficiency and coefficient of determination

Amplification efficiency and coefficient of determination (R^2^) were simultaneously calculated from standard curves generated to determine detection limit and were compared in singleplex and triplex qPCR assays. Acceptable parameters according to Bustin et al. (2009) correspond to an amplification efficiency of 90-110% and an average R^2^ value close to 0.99.

#### 2.8.2 Analytical sensitivity: Limit of detection

To determine the LOD for singleplex and triplex qPCR reactions, standard curves were constructed using 10-fold dilution series of genomic DNA from pure colonies of *N. parvum* (isolate NpALM2), *B. dothidea* (isolate BdALM2), and *Diplodia seriata* (isolate DsALM1) diluted into MilliQ sterile water or 5 ng/ml DNA solutions extracted from various matrices: subcortical tissue of almond, avocado, blueberry and grapevine plants, respectively, as well as soil and rainwater, previously confirmed to be free of *Botryosphaeriaceae* species. For triplex qPCR standard curve, 1 ng of each fungal species were mixed, and 10-fold diluted. Each standard point (1 ng to 1 fg) was analyzed in ≥ 3 replicates (three replicates were amplified for 1, 10^-1^ and 10^-2^ ng, and six replicates for 10^-3^, 10^-4^ and 10^-5^ ng standard points) and linear regression between Cq and logarithmic DNA concentration performed for quantification. Each qPCR assay was repeated at least three times.

#### 2.8.3 Asymmetric limit of detection

To determine the capacity to detect small amounts of a fungal species in the presence of high amount(s) of another, the asymmetric limit of detection (LODasym) was calculated. To do that, low amounts (10^-4^ ng of gDNA) of one of each fungal target were amplified in the presence of high amount (1 ng of gDNA) of the other two fungal species. Ten qPCR replicates were tested for each target (*N. parvum*, *B. dothidea* and *D. seriata*), respectively.

#### 2.8.4 Analytical specificity (inclusivity/exclusivity)

The specificity of the designed probes and primers was validated through both *in silico* analyses and experimental tests, as detailed in Romero-Cuadrado et al. (2023). The specificity of the new triplex-qPCR method was further evaluated using DNA from fungi within the *Botryosphaeriaceae* family isolated from almond trees and other crops including avocado, blueberry and grapevine (n=94). This included 24 *B. dothidea*, 22 N*. parvum* and 48 isolates from another *Botryosphaeriaceae* species. Additionally, 23 DNA from non-*Botryosphaeriaceae* fungi were used as negative controls (**Supplementary Table S1**).

Inclusivity and exclusivity were verified by making different DNA mixtures, including and excluding target DNAs. The first mixture contained 0.5 ng of gDNA from *Lasiodiplodia theobromae*, *Neofusicoccum mediterraneum*, *Dothiorella iberica* and *B. dothidea*, respectively. The second mixture contained an equivalent amount of gDNA from *N. parvum*, *L. theobromae*, *N. mediterraneum*, *Do. iberica* and *B. dothidea*. Finally, the third mixture contained gDNA from *N. parvum*, *L. theobromae*, *N. mediterraneum* and *Do. iberica*. The same assay was used to assess cross-talk in the triplex qPCR reactions, evaluating the overlap of adjacent detection channels during the amplification of different targets, resulting in false-positive detection. To examine this, the first DNA mixture was used to identify potential cross-talk in *N. parvum* detection with HEX fluorophore; and the third DNA mixture was used to test potential cross-talk in the detection of *B. dothidea* with FAM fluorophore. Four technical replicates were amplified for each DNA mixture. Samples detected with HEX fluorophore with Cq values >36 were suspected to be false positives due to cross-talk and require an additional singleplex qPCR for *N. parvum* detection (Np qPCR) or duplex qPCR for the simultaneous detection of *N. parvum* and *Neofusicoccum* spp. (Np/NFspp) (Romero-Cuadrado et al., 2023) to confirm results.

### 2.9 Applicability

The triplex qPCR method for the detection and quantification of two pathogenic *Botryosphaeriaceae* species along with fungi of the *Botryosphaeriaceae* family was applied for the accurate diagnosis of almond, avocado, blueberry and grapevine diseased plants from cropping fields, as well as for the preventive detection of these pathogens in asymptomatic plant material from almond nurseries. In addition, it has been verified whether fungal species of the *Botryosphaeriaceae* family could be detected and quantified in cropping soils collected from almond, avocado, blueberry and grapevine production fields and from rainwater collected from an almond orchard where *Botryosphaeriaceae* infection was previously confirmed. Finally, the qPCR was used to determine the limit of detection of aerial spores of *N. parvum* artificially inoculated on silicone covered Melinex tapes.

## 3 Results

### 3.1 Multiplex qPCR optimization

Among the various concentrations of primers, hydrolysis probes and master mixes tested in the triplex qPCR, the highest sensitivity and efficiency were achieved using a final concentration of 300 nM for primers and 200 nM for probes for each target, using the iQ Multiplex Powermix, BioRad.

### 3.2 Efficiency and coefficient of determination

**Figure 1:**
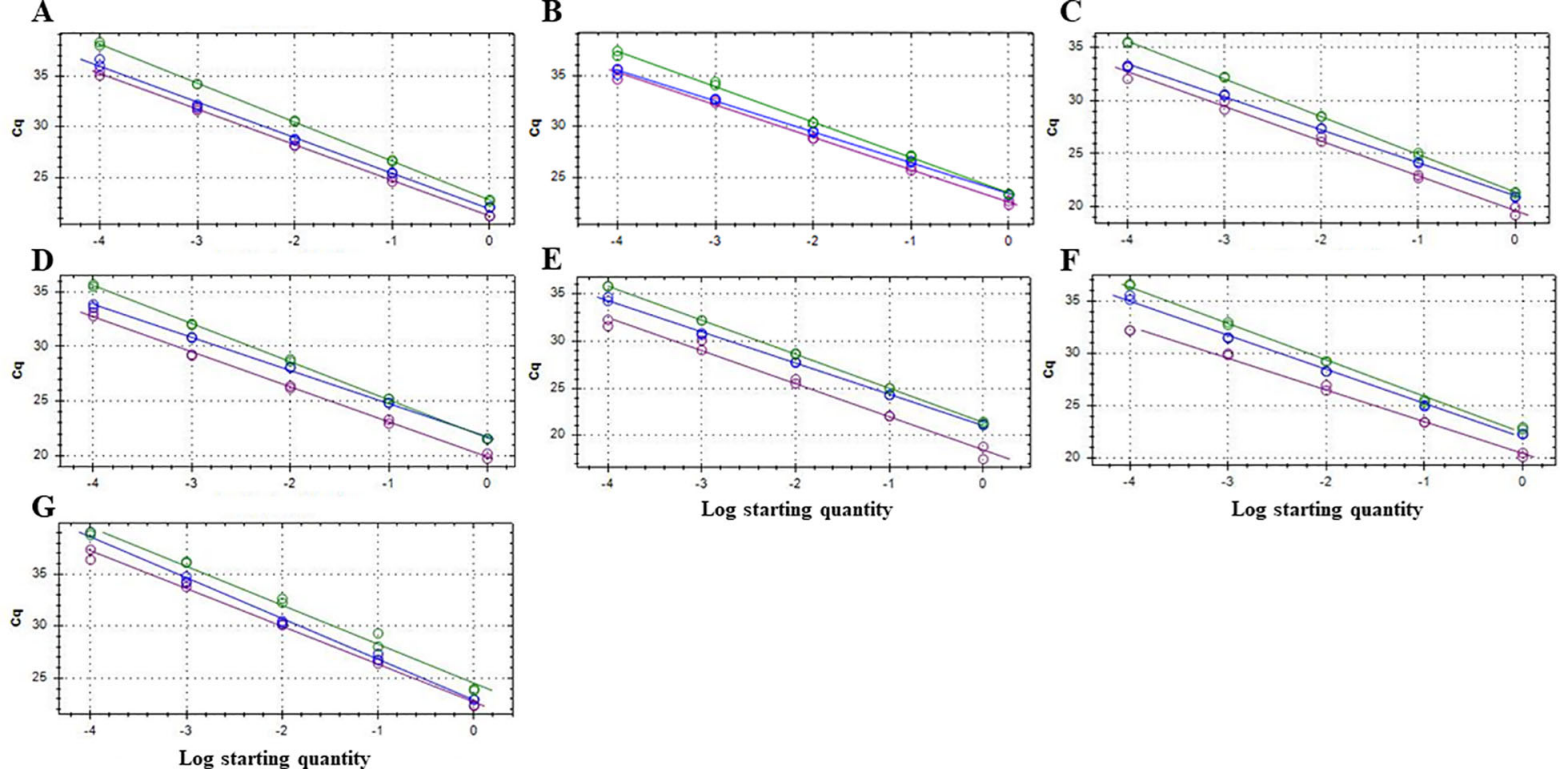
Standard curves in triplex qPCR for the quantitative and simultaneous detection of *Botryosphaeria dothidea* (detected with FAM fluorophore, green line), *Neofusicoccum parvum* (HEX fluorophore, blue line) and *Botryosphaeriaceae* family (Cy5 fluorophore, purple line) in different matrices: MilliQ sterile water **(A)**, and 5 ng/ml of DNA extracted from the subcortical tissue of almond **(B)**, avocado **(C)**, blueberry **(D)** and grapevine **(E)** plants, respectively, as well as soil **(F)** and rainwater **(G)**. Ten-fold serial dilutions of a mixture of genomic DNA from pure colonies of *B. dothidea* BdALM2 isolate, *N. parvum* NpALM2 isolate and *Diplodia seriata* DsALM2 isolate (1 to 10^-4^ ng each) were amplified in three replicates (1, 10^-1^ and 10^-2^ ng standard curve points) and six replicates (10^-3^ and 10^-4^ ng standard curve points). Each reaction was repeated at least three times.

The efficiency and the R^2^ coefficient in singleplex and triplex qPCR reactions were between 90-110%, and around 0.99, respectively, providing a good confidence (**Table 1**). The qPCR efficiencies and R^2^ coefficients of the triplex qPCR were also within the established standards when different matrices were used to construct standard curves, such as DNA from subcortical tissue of healthy plant material, soil and rainwater (**Figure 1**; **Table 2**).

**Table 1:** Amplification efficiencies and coefficient of determination (R^2^) of the singleplex and triplex qPCR reactions, calculated by CFX Master software, Bio-Rad.

Efficiency		Coefficient of determination (R^2^)		TaqMan dye	Target
Singleplex	Triplex	Singleplex	Triplex		
93.2	92.0	0.994	0.989	FAM	*Botryosphaeria dothidea*
95.9	105.5	0.989	0.981	HEX	*Neofusicoccum parvum*
103.6	107.5	0.994	0.986	Cy5	Family *Botryosphaeriaceae*

Replicates at each level n≥3.

**Table 2:** Amplification efficiencies and coefficient of determination (R^2^) of the triplex qPCR reactions using different matrices.

Matrices							TaqMan Dye	Target
MilliQ Water	Almond	Avocado	Blueberry	Grapevine	Soil	Rain water		
Efficiency (%)								
90.1	94.7	90.9	93.5	89.8	93.8	91.0	**FAM**	*Botryosphaeria dothidea*
92.9	110.2	109.7	111.0	100.5	102.8	90.2	**HEX**	*Neofusicoccum parvum*
93.1	107.3	102.0	105.1	93.0	112.3	89.6	**Cy5**	Family *Botryosphaeriaceae*
Coefficient of Determination (R^2^)								
0.999	0.998	0.999	0.999	1.000	0.996	0.988	**FAM**	*Botryosphaeria dothidea*
0.996	0.999	0.999	0.998	0.999	0.996	0.996	**HEX**	*Neofusicoccum parvum*
0.999	0.996	0.993	0.997	0.990	0.995	0.994	**Cy5**	Family *Botryosphaeriaceae*

Standard curves were constructed with serial dilutions of gDNA from *Botryosphaeria dothidea* BdALM2, *Neofusicoccum parvum* NpALM2 and *Diplodia seriata* DsALM2 isolates (see Material and methods) diluted in 5 ng/ml of DNA extracted from the subcortical tissue of almond, avocado, blueberry and grapevine healthy plants, soil and rainwater, respectively. Replicates at each level n≥3.

### 3.3 Analytical sensitivity: LOD and asymmetric LOD

**Figure 2:**
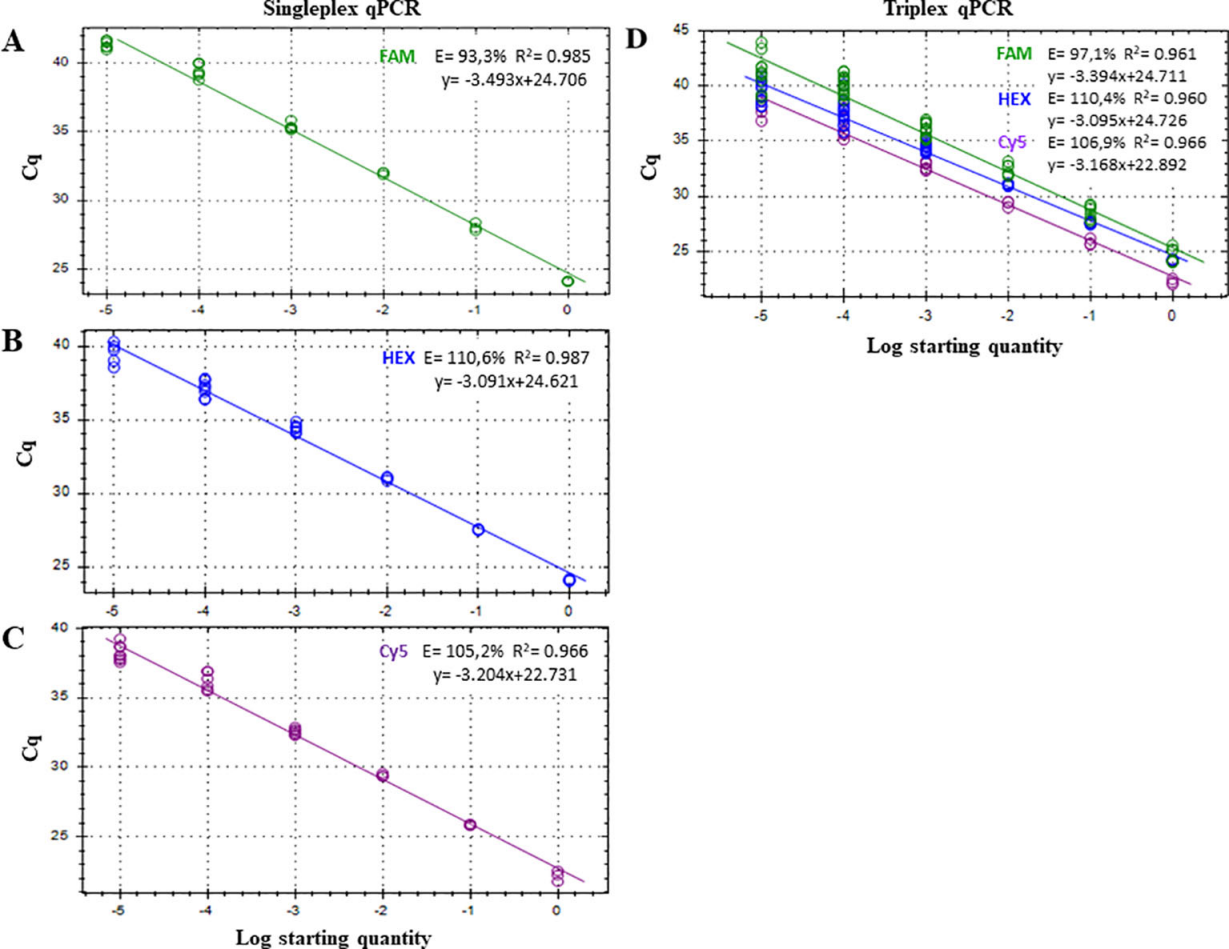
Standard curves for the quantitative detection of **(A)** *Botryosphaeria dothidea*, detected with FAM fluorophore; **(B)** *Neofusicoccum parvum*, detected with HEX fluorophore; and **(C)** *Botryosphaeriaceae* family, detected with Cy5 fluorophore under singleplex and triplex **(D)** qPCR assays. Serial dilutions of gDNA from pure colonies of *B. dothidea*, *N. parvum* and *Diplodia seriata* were amplified in three (1 ng to 10 pg) or six (1 pg to 10 fg) replicates. Efficiency (E), coefficient of determination (R^2^), and regression equations of standard curves are shown for each qPCR reaction. Each reaction was repeated at least three times.

**Figure 3:**
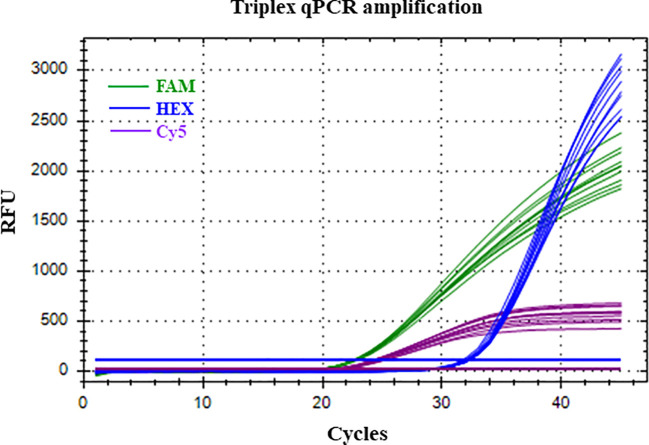
Successful amplification of *Neofusicoccum parvum* present in low concentration (100 fg of gDNA) with HEX fluorophore, in the presence of high amounts (1 ng of gDNA) of *Botryosphaeria dothidea* (FAM fluorophore) and *Diplodia seriata* (Cy5 fluorophore) respectively. Replicates at each level n= 10). RFU, Relative fluorescent units.

The LOD of the triplex qPCR was 10 fg of genomic DNA for the three fungal targets (**Figure 2**; **Table 3**). In addition, the technique successfully detected 100 fg of gDNA for any fungal target in a mixture where the other two are in abundance (**Figure 3**; **Table 4**). Therefore, there was no competition due to the concurrent presence of other targets, and the LOD and the asymmetric LOD were similar.

**Table 3:** Comparison of the limit of detection (LOD) between singleplex and triplex qPCR using different concentrations of a mix of serially diluted genomic DNA of *Botryosphaeria dothidea*, *Neofusicoccum parvum* and *Diplodia seriata*.

gDNA (ng)	Mean Cq ± SE^a^					
	Singleplex qPCR			Triplex qPCR		
	FAM	HEX	Cy5	FAM	HEX^b^	Cy5
1	24.1 ± 0.03	24.12 ± 0.04	22.19 ± 0.23	24.08 ± 6 0.1	24.26 ± 0.02	22.27 ± 0.14
10^-1^	28.06 ± 0.18	27.55 ± 0.00	25.85 ± 0.04	27.86 ± 0.14	27.55 ± 0.10	25.85 ± 0.17
10^-2^	31.99 ± 0.07	31.02 ± 0.07	29.43 ± 0.07	31.67 ± 0.18	31.05 ± 0.05	29.33 ± 0.16
10^-3^	35.37 ± 0.18	34.44 ± 0.11	32.56 ± 0.09	35.20 ± 0.13	34.24 ± 0.13	32.77 ± 0.14
10^-4^	39.44 ± 0.21	37.22 ± 0.21	36.19 ± 0.26	38.94 ± 0.15	37.40 ± 0.36	35.95 ± 0.24
10^-5^	41.39 ± 0.12	39.52 ± 0.32	38.43 ± 0.22	40.15 ± 0.42	39.23 ± 0.47	38.10 ± 0.32

a Cq, quantification cycle; Data are the mean of 3 to 6 replicates ± standard error.

b Detection of *Neofusicoccum parvum* with Ct>36 require an additional analysis by singleplex or duplex qPCR (Romero-Cuadrado et al., 2023) to discard false positive detection due to cross-talk.

**Table 4:** Determination of the asymmetric limit of detection (LOD_asym_) in the triplex qPCR.

Triplex qPCR condition	gDNA amount^a^			Mean Cq ± SE^b^		
	*Bd*	*Np*	BOT	*Bd* (FAM)	*Np* (HEX)	BOT (Cy5)
LOD_asym_ *Bd*	100 fg	1 ng	1 ng	39.36 ± 0.23	23.91 ± 0.05	22.40 ± 0.06
LOD_asym_ *Np*	1 ng	100 fg	1 ng	23.74 ± 0.05	35.35 ± 0.07	22.94 ± 0.09
LOD_asym_ BOT	1 ng	1 ng	100 fg	23.64 ± 0.04	23.93 ± 0.04	23.17 ± 0.07

a *Bd, Botryosphaeria dothidea* BdALM2; *Np, Neofusicoccum parvum* NpALM2; BOT, *Diplodia seriata* DsALM2.

b Cq, quantification cycle. Data are the mean of 10 replicates ± standard error.

### 3.4 Inclusivity/exclusivity

The triplex qPCR demonstrated exclusivity and inclusivity by not amplifying DNA from fungal species not included and successfully amplifying when they were added to the qPCR reaction (**Table 5**). However, it is important to consider that cross-talk occurs in the detection of *N. parvum* with the HEX-labeled probe in the triplex qPCR. Therefore, samples with a Cq value of 36 or higher in the detection of *N. parvum* are considered uncertain. A negative result for *Botryosphaeriaceae* family in this sample (no detection with Cy5 fluorophore) implies negativity for *N. parvum*, while a positive result for *Botryosphaeriaceae* family and a Cq>36 for HEX fluorophore necessitates verification of detection reliability via singleplex or duplex qPCR. No cross-talk reaction was observed for the detection of *B. dothidea* with the triplex qPCR.

**Table 5:** Inclusivity and exclusivity of the triplex qPCR.

Fungal species in DNA mix^a^	Mean Cq ± SE^b^		
	*Bd*	*Np*	BOT
*Lt* + *Nm* + *Doi* + *Bd*	21.36 ± 0.13	-^c^	23.01 ± 0.05
*Np* + *Lt* + *Nm* + *Doi* + *Bd*	21.20 ± 0.14	24.58 ± 0.07	22.31 ± 0.09
*Np* + *Lt* + *Nm* + *Doi*	–	24.88 ± 0.09	23.17 ± 0.02

a Genomic DNA (0.5 ng each) from *Lasiodiplodia theobromae* (*Lt*); *Neofusicoccum mediterraneum* (*Nm*); *Dothiorella iberica* (*Doi*); *Botryosphaeria dothidea* (*Bd*); *Neofusicoccum parvum* (*Np*).

b Data were the mean of the amplification quantity (Cq) ± standard error of four replicates; - no detection. *Bd: B. dothidea* detected with FAM fluorophore; *Np: N. parvum* detected with HEX fluorophore, BOT: *Botryosphaeriaceae* family detected with Cy5 fluorophore.

c Samples with Cq values >36, suspected to be false positives due to cross-talk, require an additional duplex qPCR for the simultaneous detection of *Neofusicoccum parvum* and *Neofusicoccum* spp. (Romero-Cuadrado et al., 2023) for results confirmation.

### 3.5 Applicability

The developed triplex qPCR protocol was applied for accurate diagnosis of diseased plants in almond, avocado, blueberry and grapevine field crops as well as for the preventive detection of *Botryosphaeriaceae* fungi in asymptomatic propagation material from almond nurseries. It was further applied for the detection of *Botryosphaeriaceae* species in crop soils, rainwater and artificially inoculated trapped spores.

#### 3.5.1 Almond

In the analysis of 26 symptomatic almond trees from production fields, 12 tested positive for *Botryosphaeriaceae* family by qPCR detection. Specifically, ten were detected as positive for *B. dothidea* and two for *N. parvum*. One of the diseased trees showed a mixed infection with *B. dothidea* and *M. phaseolina*, confirmed through PDA culture isolation and subsequent sequencing of hyphal-typed isolates. Among the six asymptomatic trees, two showed positive results for *Botryosphaeriaceae*, one of them also for *B. dothidea* (**Supplementary Table S3**).

In the analysis of 30 asymptomatic grafting scions from mother plant trees at an almond nursery conducted in October 2022, all samples tested negative for the three fungal targets. In the analysis performed in June 2023 in the same nursery, three out of 15 grafting scions from mother plant trees tested positive for *Botryosphaeriaceae* detection while being negative for *B. dothidea* and *N. parvum*. Tissue culture on PDA plates and subsequent sequencing confirmed *D. seriata* infection in all three positive samples and *Dothiorella viticola* in one scion, which exhibited a double infection. Among the 10 GxN-15 rootstock plants analyzed, five were positive for *Botryosphaeriaceae* detection and negative for the other two fungal targets. Tissue culture and subsequent sequencing confirmed the presence of *M. phaseolina* in the secondary roots of two plants and in the main root of another (**Supplementary Table S3**).

*Botryosphaeriaceae* fungi were detected in 44.8% (13 out of 29) of symptomatic almond samples and in 13.6% (11 out of 81) of asymptomatic almond samples by the triplex qPCR assay.

#### 3.5.2 Avocado

All 120 asymptomatic scions collected from ‘Hass’ and ‘Reed’ avocado varieties in October 2022 resulted negative for *Botryosphaeriaceae* detection and plate culture isolation. From the 100 scions collected from ‘Hass’ avocado trees in October 2023, 13 tested positive for *Botryosphaeriaceae*, with 11 of them also positive for *N. parvum*. (**Supplementary Table S3**). Out of these 11 samples, 4 exhibited Ct values above 36 and tested positive upon confirmation through duplex qPCR for specific detection of *N. parvum* and *Neofusicoccum* spp. Plate culture was deemed unsuitable for isolating *Botryosphaeriaceae* species due to high *Alternaria* spp. content in the scions, with the exception of one sample positive for *Botryosphaeriaceae* from which *Lasiodiplodia* spp. was identified (sample Avo3) and a sample positive for *Botryosphaeriaceae* and *N. parvum* from which infection with *N. parvum* was confirmed by plate culture isolation (sample Avo5) (**Supplementary Table S3**).

*Botryosphaeriaceae* fungi were detected by triplex qPCR in 100% (8 out of 8) of symptomatic avocado samples, and in 0% (0 out of 120) and 5% (5 out of 100) of asymptomatic avocado samples analyzed in 2022 and 2023, respectively.

#### 3.5.3 Blueberry

Out of the 14 diseased blueberry plants uprooted from production fields, 4 tested positive for *Botryosphaeriaceae* (28.6%), with one also positive for *N. parvum*. Subsequent species identification within *Botryosphaeriaceae* confirmed *N. parvum* infection and revealed infection by *L. theobromae* in the remaining three positive plants. The other diseased plants, where *Botryosphaeriaceae* was not detected, were predominantly infected by *Neopestalotiopsis rosae* (data not shown). The asymptomatic blueberry plant resulted negative for detection of the three fungal targets (**Supplementary Table S3**).

#### 3.5.4 Grapevine

All 10 collected diseased grapevine plants tested positive for *Botryosphaeriaceae* detection (100%), with one of them also showing positive amplification for *N. parvum*. Tissue culture on PDA confirmed the infection by *N. parvum* in this plant and by *D. seriata* in eight of the *Botryosphaeriaceae*-positive plants, while the *Botryosphaeriaceae* species present in the remaining plant could not be identified by this method. Among the two asymptomatic plants, one tested positive for *Botryosphaeriaceae* and *N. parvum* probes, while the other yielded negative results for any target (**Supplementary Table S3**).

#### 3.5.5 Agreement between triplex qPCR and plate culture and further sequencing detection methods

**Figure 4:**
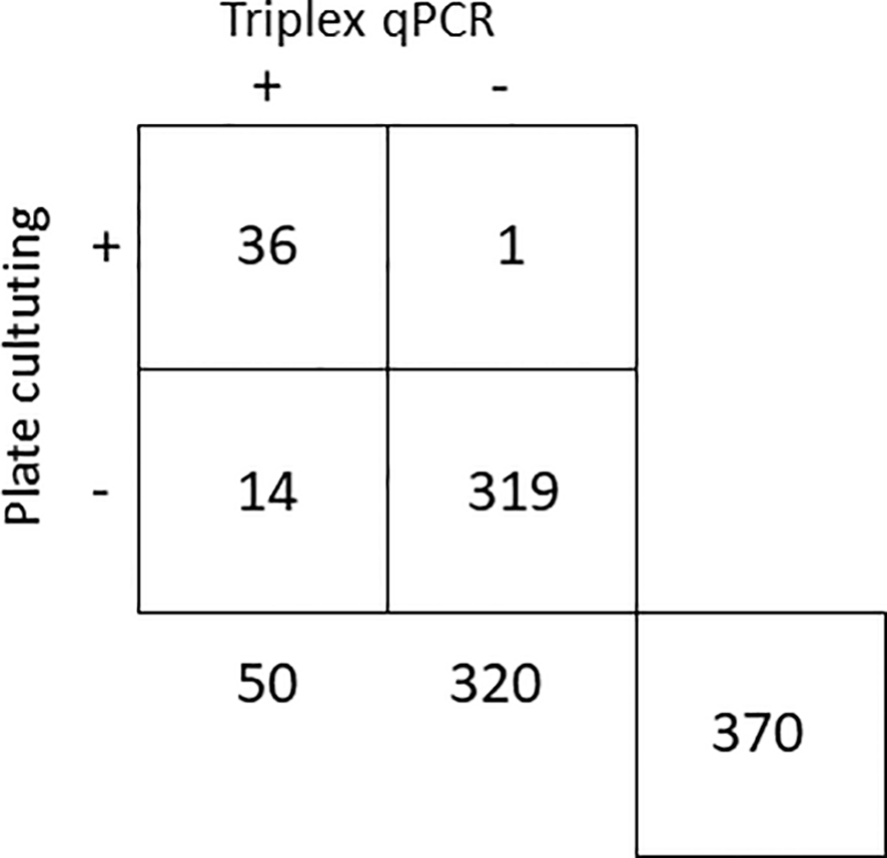
Comparison of triplex qPCR and plate culturing methods for the detection of *Botryosphaeriaceae* fungi in 370 samples from almond, avocado, blueberry and grapevine symptomatic and asymptomatic plants.

In the analyses of 370 samples from almond, avocado, blueberry and grapevine plants, a substantial agreement was found between the two detection methods, with a Kappa index of 0.8052 ± 0.0512 (Landis and Koch, 1997). Out of the 370 samples analyzed, 36 tested positive and 319 tested negative by both methods. Fourteen samples were positive by qPCR but not by plate culturing, and only one resulted negative by qPCR and positive by plate culturing and further sequencing (**Figure 4**). Of 75 symptomatic samples assessed, 35 (46.7%) tested positive for infection by any of the three fungal targets. Among the 295 asymptomatic samples, 17 (5.8%) were found to be positive for any of the three fungal targets.

#### 3.5.6 Soil

The developed triplex qPCR protocol was further applied for the detection of *Botryosphaeriaceae* species in crop soils (**Supplementary Table S4**). Out of 10 soil samples analyzed from almond orchards, seven yielded positive results for *Botryosphaeriaceae* family. Among these positive samples, two tested positive for *B. dothidea*, while another five were positive for *M. phaseolina*, as confirmed by plate culturing.

Of the five soils collected from avocado orchards, all tested positive for the *Botryosphaeriaceae* family. In addition, one of these samples also tested positive for *N. parvum*, confirmed by Np/NFspp duplex qPCR.

Out of three soil samples collected from blueberry orchards, two were confirmed to be positive for *Botryosphaeriaceae* family. Furthermore, these two soils were infected by *M. phaseolina* as confirmed by plate culturing.

Of the nine soils collected from grapevine orchards, only one was positive for the detection of *Botryosphaeriaceae* family. This sample also tested positive for *B. dothidea* and for the detection of *N. parvum*, the latter confirmed by Np/NFspp duplex qPCR.

Average inoculum densities of *Botryosphaeriaceae* in the analyzed soils ranged from 0.44 pg DNA/g soil to 553 pg DNA/g soil.

#### 3.5.7 Rainwater

The DNeasy PowerSoil Pro Kit demonstrated superior efficiency compared to the other two commercial kits tested for DNA extraction. All rainwater samples collected from the almond orchard (next to 5 trees in 4 different months) tested positive for *Botryosphaeriaceae* family, except for R_M_Sol 4-7 collected in February. Among these samples, *B. dothidea* was detected in all 5 tree locations in November or December, but not in January and February. One sample yielded positive for *N. parvum* and another for all three fungal targets (**Supplementary Table S4**), both confirmed as *N. parvum* positive by Np-NFspp duplex-qPCR. Average inoculum densities of *Botryosphaeriaceae* in the collected rainwater ranged from 0.83 pg DNA/L rainwater to 210 pg DNA/L rainwater.

#### 3.5.8 Trapped spores

**Figure 5:**
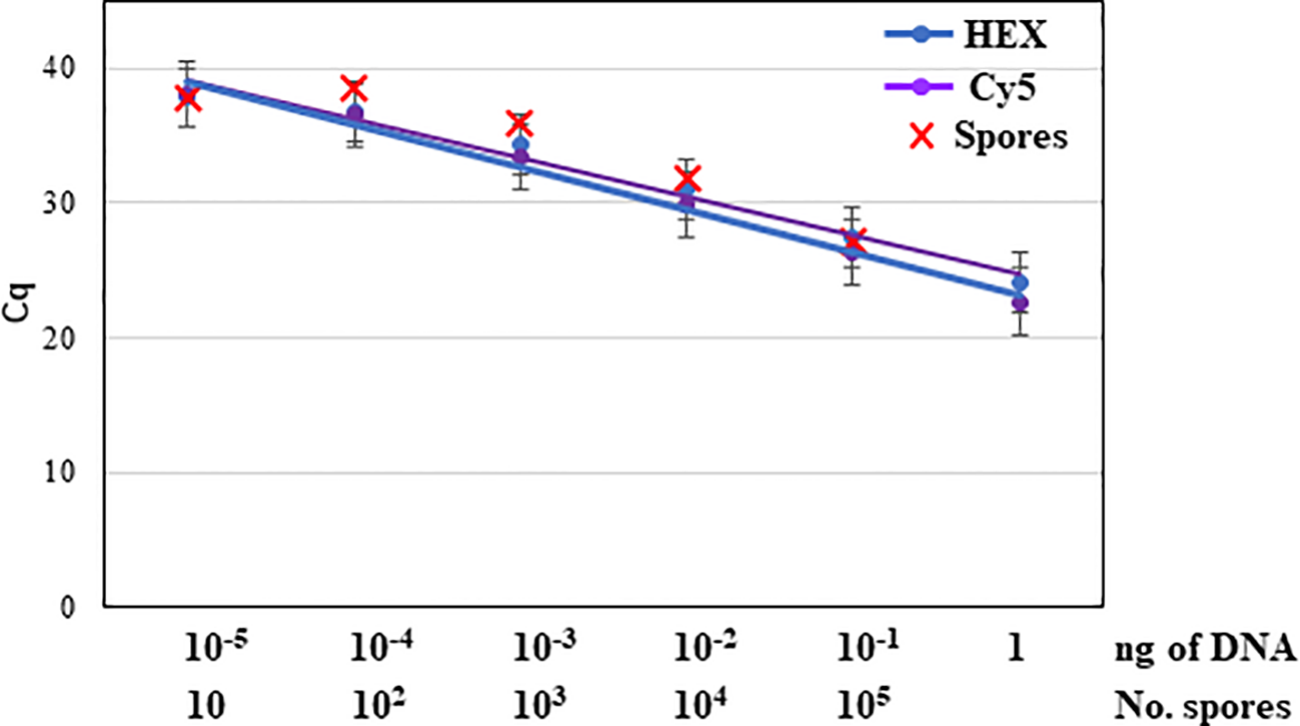
Detection of serial dilutions (10 to 10^5^) of trapped spores from *Neofusicoccum parvum* isolate NpALM2 artificially inoculated onto glass microscope slides covered with Melinex tapes coated with silicone oil by triplex qPCR. Nine repetitions per dilution were included.

Among the two commercial kits tested for DNA extraction, the DNeasy PowerSoil Pro Kit proved to be more efficient. Out of 50 µl of total DNA extracted from the spore trap, 5 µl were used as template for qPCR. The limit of detection of *N. parvum* trapped spores by the triplex qPCR was 10 conidia, as slides inoculated with 100 conidia were successfully amplified by triplex qPCR and detected with HEX and Cy5 fluorophores. Slides inoculated with 10 conidia were also amplified, although the repeatability was poor, with only 20% of repetitions yielding an amplification signal (**Figure 5**).

## 4 Discussion

*Botryosphaeriaceae* fungal diseases have increased in recent decades, attributed to the ban on chemical fungicides mandated by the EU, the intensification of crops aimed at increasing productivity, the globalization of the nursery plant market (Vettraino et al., 2017) and the escalating temperatures and drought associated with climate change (Batista et al., 2023; Fernández et al., 2023; Velásquez et al., 2018). A new multiplex quantitative real-time polymerase chain reaction (qPCR) assay was developed for the simultaneous detection and quantification of *B. dothidea*, *N. parvum* and any species within the *Botryosphaeriaceae* family in symptomatic and asymptomatic plants and environmental samples (soil, rainwater and trapped spores). This powerful molecular tool enables an accurate and rapid diagnosis of wood diseases affecting economically important agricultural crops such as almond, avocado, blueberry and grapevine, which may exhibit undistinguishable symptoms caused by woody pathogens. A correct diagnosis is one of the most effective strategies for controlling plant diseases and minimizing yield losses (Venbrux et al., 2023). Moreover, knowing the causal agent allows for an accurate application of control measures, saving cost and protecting the environment.

The triplex qPCR protocol has a limit of detection of 10 fg gDNA for any of the three fungal targets. This sensitivity is consistent with those achieved in three independent duplex qPCR protocols we developed in previous work for the simultaneous detection of *N. parvum* and the *Neofusicoccum* genus, *N. parvum* and the *Botryosphaeriaceae* family and, *B. dothidea* and the *Botryosphaeriaceae* family (Romero-Cuadrado et al., 2023). Multiplex detection offers many advantages compared to duplex or single target detection, including high throughput and reduced cost. However, the use of three fluorescent dyes may result in cross-talk detection and, consequently, the emergence of false-positive results. This is particularly observed in the detection of *N. parvum* with HEX fluorophore. Therefore, for reliable results, detections of *N. parvum* above Ct 36 (i.e., 10^-4^ ng DNA) should be confirmed by simplex or duplex qPCR in a parallel assay (Romero-Cuadrado et al., 2023).

One of the main advantages of the designed qPCR protocol is its high sensitivity compared to conventional and nested PCR previously reported (Avenot et al., 2022; Cheng-nan et al., 2016; Ni et al., 2012; Ridgway et al., 2011). In addition, the qPCR assay is high throughput and more accurate because it does not require of subsequent analyses, thereby preventing potential cross contamination and false positive detections. Furthermore, the designed qPCR uses TaqMan probes, which have demonstrated to be more sensitive than protocols using SYBR Green chemistry (Luo et al., 2017; Haidar et al., 2020; Billones-Baaijens et al., 2018).

The high sensitivity of the multiplex qPCR enables the early detection of these fungal pathogens in asymptomatic nursery plant material, serving as a preventive control. In many cases, when the plant is infected during the nursery process, the disease typically manifests in the first year post-plantation (Moral et al., 2019). This leads to the decline and death of the recently planted plant, which must be replaced, significantly increasing the cost of establishing a new plantation. Therefore, this qPCR protocol could be preventively applied for the analyses of all stages of plant production process in nurseries, avoiding the “silent” introduction of these pathogenic fungi into production fields and their subsequent spread. The developed triplex qPCR protocol was able to detect *Botryosphaeriaceae* species in asymptomatic material from almond nurseries. Despite not observing any incidence (0%) in the October 2022 survey, *D. seriata* and *Do. viticola* were detected in three out of 30 grafting scions from mother plants analyzed in October 2023. Additionally, *M. phaseolina* was detected in the main and secondary roots of five out of 10 analyzed GxN-15 uprooted rootstocks. The origin of these infections remains unknown and should be explored through a comprehensive analysis of all nursery almond production steps. The infection of roots by *M. phaseolina* could originate from either the plant material used as rootstock or the soil where the plant was established before grafting. Therefore, conducting an exhaustive analysis of both the plant material and the substrate in nurseries and production fields by a sensitive and specific qPCR detection method is crucial. These results confirm that almond nursery material can serve as an external inoculum source for *Botryosphaeriaceae* species. Previous studies have also highlighted nursery material as a potential source of *Botryosphaeriaceae* infection in several crops (Billones-Baaijens et al., 2013c; Gramaje et al., 2022; Polizzi et al., 2023; Ramírez-Gil and Osorio, 2021; Sosso et al., 2021; Tennakoon et al., 2018), including almond (Luo et al., 2024). Additionally, singleplex qPCR protocols have been used to detect some genera of *Botryosphaeriaceae* and other canker-causing pathogens in asymptomatic samples from almond, walnut, pistachio and dried plums production fields (Luo et al., 2019).

The growing demand for almond varieties to establish new almond production fields has necessitated importing plant material from other countries in many cases. In this sense, this qPCR protocol could serve as a preventive measure to analyze the phytosanitary status of the imported material. This new multiplex qPCR molecular tool could be of significant interest to regulatory authorities and diagnostic laboratories, allowing the testing of multiple pathogens in a single tube, thereby reducing costs and rendering the technique highly cost-effective.

A high agreement (k = 0.8052 ± 0.0512) was observed between triplex qPCR and the traditional plate culturing detection methods in the analysis of plant samples, and a high percentage (95.94%) of coincidental results was obtained. The triplex qPCR method showed to be more sensitive that the traditional plate culturing, as 14 samples (3.78%) were detected by qPCR but not by the traditional isolation method. On the contrary, only one sample (0.27%) was detected as positive for *Botryosphaeriaceae* infection and not detected by qPCR, probably due to problems inherent to sampling, manipulation or inhibition of the qPCR reaction. Among symptomatic plant samples, 46.7% tested positive for any of the three fungal targets. The rest of symptoms could be attributed to mechanical damage or caused by infections from other pathogenic microorganisms. On the contrary, almost 6% of asymptomatic plant samples were found to be infected by *Botryosphaeriaceae* species, demonstrating the danger of latent infections.

Beyond detection, qPCR facilitates pathogen quantification. The developed triplex qPCR protocol was further applied for the detection and quantification of *Botryosphaeriaceae* species in crop soils and rainwater, and also demonstrated the ability to detect as less as 10 trapped spores of *Botryosphaeriaceae* fungi. This capability allows the establishment of action thresholds in the field, indicating the pathogen inoculum level that would require a treatment (Almquist et al., 2016). Furthermore, this approach would reduce the frequency of chemical pesticide applications, contributing to a more sustainable control of the disease (Thiessen et al., 2017). This qPCR tool could also be applied in breeding programs to screen the susceptibility of different crop varieties to *Botryosphaeriaceae* species, by quantifying the evolution of inoculum levels in artificially inoculated plants, even before symptom manifestation.

Since mixed *Botryosphaeriaceae* infections occur in nature (Úrbez-Torres et al., 2006), another advantage of the designed qPCR is its ability to detect multiple targets in the same reaction. Therefore, in a single test, the qPCR could detect *B. dothidea*, the most abundant *Botryosphaeriaceae* species in almond crops in Spain (Agustí-Brisach et al., 2020a; Antón Domínguez et al., 2023), *N. parvum*, the most aggressive species in almond and other economically important crops (Aiello et al., 2022; Billones-Baaijens et al., 2013a; Carlucci et al., 2013; Chen et al., 2014; Fernández et al., 2023; Olmo et al., 2016; Úrbez-Torres, 2011; Valencia et al., 2019), and any species within the *Botryosphaeriaceae* family, even when one of the fungus has lower abundance compared to the other two targets, as we have demonstrated by the asymmetric LOD.

Additionally, fungal species of the *Botryosphaeriaceae* family can be detected by the multiplex qPCR in environmental samples like crop soils, rainwater and trapped spores. This provides a powerful tool to determine potential sources of inoculum and a better understanding of the epidemiology of *Botryosphaeriaceae* fungi. Soil may serve as a source of inoculum for soil-borne species such as *M. phaseolina* (Márquez et al., 2021), or even for aerial species that survive in pruning debris on the ground (Billones-Baaijens et al., 2013b). In addition, the triplex qPCR protocol was able to detect as less as 10 spores of *N. parvum* artificially inoculated in sticky tapes, although in same cases as less as 1 spore could be detected. Understanding the abundance and seasonal distribution of fungal spores in the air is crucial for timely application of the appropriate control measures and for developing an effective schedule for implementing phytosanitary measures. On the other hand, triple detection enables understanding the relationship between these fungi based on environmental conditions, in soil, water, and plant tissue. Other studies have used qPCR protocols for detecting and quantifying *Botryosphaeriaceae* spores from the environment (Billones-Baaijens et al., 2018; Hernández and Kc, 2024; Luo et al., 2019; Pouzoulet et al., 2017), all using singleplex qPCR reactions.

Currently, there is no cure for plants infected by *Botryosphaeriaceae* (Gramaje et al., 2018), and, furthermore, no woody crop variety resistant to *Botryosphaeriaceae* is currently known. Therefore, preventive detection stands out as one the most effective management strategies. The newly developed triplex qPCR assay enables accurate diagnosis of symptomatic plants, facilitating the implementation of appropriate control measures. Moreover, this powerful tool can detect *Botryosphaeriaceae* fungi in asymptomatic nursery plant material, preventing the introduction and spread of these pathogens in production fields. Additionally, its capacity to detect *Botryosphaeriaceae* fungi in environmental samples is valuable for identifying inoculum sources and quantifying inoculum levels in crop environments, contributing to get a precise phytosanitary application schedule, thereby reducing production costs and preserving the environment. In summary, the new triplex qPCR protocol constitutes a sustainable and preventive control method for *Botryosphaeriaceae* fungal diseases affecting economically important woody crops.

## Data Availability

The original contributions presented in the study are included in the article/supplementary material. Further inquiries can be directed to the corresponding author.

## References

[B1] Agustí-Brisach C. León M. García-Jiménez J. Armengol J. (2015). Detection of grapevine fungal trunk pathogens on pruning shears and evaluation of their potential for spread of infection. Plant Dis. 99, 976–981. doi: 10.1094/PDIS-12-14-1283-RE 30690978

[B2] Agustí-Brisach C. Moldero D. Raya M. D. Lorite I. J. Orgaz F. Trapero A. (2020a). Water stress enhances the progression of branch dieback and almond decline under field conditions. Plants-Basel 9, 1213. doi: 10.3390/plants9091213 32947913 PMC7570136

[B3] Aiello D. Bregant C. Carlucci A. Guarnaccia V. Gusella G. Linaldeddu B. T.. (2023). Current status of Botryosphaeriaceae species in Italy: Impacts on agricultural crops and forest ecosystems. Phytopathol. Mediterr. 62, 381–412. doi: 10.36253/phyto-14711

[B4] Aiello D. Guarnaccia V. Costanzo M. B. Leonardi G. R. Epifani F. Perrone G.. (2022). Woody canker and shoot blight caused by *Botryosphaeriaceae* and *Diaporthaceae* on mango and litchi in Italy. Horticulturae 8, 330. doi: 10.3390/horticulturae8040330

[B5] Almquist C. Persson L. Olsson A. Sundstrom J. Jonsson A. (2016). Disease risk assessment of sugar beet root rot using quantitative real-time PCR analysis of *Aphanomyces cochlioides* in naturally infested soil samples. Eur. J. Plant Pathol. 145, 731–742. doi: 10.1007/s10658-016-0862-5

[B6] Antón Domínguez B. I. López-Moral A. Raya-Ortega M. C. Lovera M. Melgar S. Roca-Castillo L. F.. (2023). Fungal pathogens associated with almond decline syndrome, an emerging disease complex in intensive almond crops in southern Spain. Plant Dis. 107, 3737–3753. doi: 10.1094/PDIS-04-23-0759-RE 37486269

[B7] Arjona-Girona I. Ruano-Rosa D. López-Herrera C. J. (2019). Identification, pathogenicity and distribution of the causal agents of dieback in avocado orchards in Spain. Spanish J. Agric. Res. 17, e1003–e10035. doi: 10.5424/sjar/2019171-13561

[B8] Avenot H. F. Jaime-Frias R. Travadon R. Holland L. A. Lawrence D. P. Trouillas F. P. (2022). Development of PCR-based assays for rapid and reliable detection and identification of canker-causing pathogens from symptomatic almond trees. Phytopathology 112, 1710–1722. doi: 10.1094/PHYTO-08-21-0351-R 35240867

[B9] Baskarathevan J. Jaspers M. V. Jones E. E. Ridgway H. J. (2013). Development of isolate-specific markers for Neofusicoccum parvum and *N. luteum* and their use to study rainwater splash dispersal in the vineyard. Plant Pathol. 62, 501–509. doi: 10.1111/j.1365-3059.2012.02675.x

[B10] Batista E. Lopes A. Alves A. (2021). What do we know about *Botryosphaeriaceae*? An overview of a worldwide cured dataset. Forests 12, 313. doi: 10.3390/f12030313

[B11] Batista E. Lopes A. Miranda P. Alves A. (2023). Can species distribution models be used for risk assessment analyses of fungal plant pathogens? A case study with three *Botryosphaeriaceae* species. Eur. J. Plant Pathol. 165, 41–56. doi: 10.1007/s10658-022-02587-7

[B12] Belair M. Restrepo-Leal J. D. Praz C. Fontaine F. Remond C. Fernandez O.. (2023). *Botryosphaeriaceae* gene machinery: Correlation between diversity and virulence. Fungal Biol. 127(5), 1010–1031. doi: 10.1016/j.funbio.2023.03.004 37142361

[B13] Billones-Baaijens R. Jones E. E. Ridgway H. J. Jaspers M. V. (2013a). Virulence affected by assay parameters during grapevine pathogenicity studies with. Botryosphaeriaceae nursery isolates. Plant Pathol. 62, 1214–12255. doi: 10.1111/ppa.12051

[B14] Billones-Baaijens R. Ridgway H. J. Jones E. E. Cruickshank R. H. Jaspers M. V. (2013c). Prevalence and distribution of *Botryosphaeriaceae* species in New Zealand grapevine nurseries. Eur. J. Plant Pathol. 135, 175–185. doi: 10.1007/s10658-012-0076-4

[B15] Billones-Baaijens R. Ridgway H. J. Jones E. E. Jaspers M. V. (2013b). Inoculum sources of *Botryosphaeriaceae* species in New Zealand grapevine nurseries. Eur. J. Plant Pathol. 135, 159–174. doi: 10.1007/s10658-012-0075-5

[B16] Billones-Baaijens R. Urbez-Torres J. R. Liu M. Ayres M. Sosnowski M. Savocchia S. (2018). Molecular methods to detect and quantify *Botryosphaeriaceae* inocula associated with grapevine dieback in Australia. Plant Dis. 102, 1489–1499. doi: 10.1094/PDIS-11-17-1854-RE 30673411

[B17] Bustin S. A. Benes V. Garson J. A. Hellemans J. Huggett J. Kubista M.. (2009). The MIQE guidelines: Minimum information for publication of quantitative real-time PCR experiments. Clin. Chem. 55, 611–6225. doi: 10.1373/clinchem.2008.112797 19246619

[B18] Carbone M. J. Gelabert M. Moreira V. Mondino P. Alaniz S. (2022). Grapevine nursery propagation material as source of fungal trunk disease pathogens in Uruguay. Front. Fungal Biol. 3. doi: 10.3389/ffunb.2022.958466 PMC10512308 37746215

[B19] Carlucci A. Cibelli F. Lops F. Raimondo M. L. (2015). Characterization of botryosphaeriaceae species as causal agents of trunk diseases on grapevines. Plant Dis. 99, 1678–16885. doi: 10.1094/PDIS-03-15-0286-RE 30699521

[B20] Carlucci A. Raimondo M. L. Cibelli F. Phillips A. J. L. Lops F. (2013). *Pleurostomophora richardsiae*, *Neofusicoccum parvum* and *Phaeoacremonium aleophilum* associated with a decline of olives in southern Italy. Phytopathol. Mediterr. 52, 517–527. Available at: http://www.jstor.org/stable/42685430.

[B21] Chen S. F. Morgan D. P. Michailides T. J. (2014). Botryosphaeriaceae and *Diaporthaceae* associated with panicle and shoot blight of pistachio in California, USA. Fungal Diversity 67, 157–1795. doi: 10.1007/s13225-014-0285-6

[B22] Cheng-nan X. Hong-jun Z. Fu-mei C. Zhi-rui J. Qing-long D. Ke-qiang C. (2016). Species-specific PCR-based assays for identification and detection of *Botryosphaeriaceae* species causing stem blight on blueberry in China. J. Integr. Agric 15, 573–579.

[B23] Doll D. A. Michailides T. J. Rolshausen P. E. (2013). Botryosphaeriaceae associated with almond trunk cankers: A threat to the almond industry? Phytopathology 103, 13–135.

[B24] English H. (1975). Relationship of *Botryosphaeria dothidea* and *Hendersonula toruloidea* to a canker disease of almond. Phytopathology 65, 114–122. doi: 10.1094/Phyto-65-114

[B25] Espinoza J. G. Briceno E. X. Chavez E. R. Úrbez-Torres J. R. Latorre B. A. (2009). Neofusicoccum spp. associated with stem canker and dieback of blueberry in Chile. Plant Dis. 93, 1187–11945. doi: 10.1094/PDIS-93-11-1187 30754575

[B26] Fernández O. Lemaitre-Guillier C. Songy A. Robert-Siegwald G. Lebrun M. H. Schmitt-Kopplin P.. (2023). The combination of both heat and water stresses may worsen Botryosphaeria dieback symptoms in grapevine. Plants-Basel 12, 753. doi: 10.3390/plants12040753 36840101 PMC9961737

[B27] Fiorenza A. Gusella G. Vecchio L. Aiello D. Polizzi G. (2023). Diversity of *Botryosphaeriaceae* species associated with canker and dieback of avocado (*Persea americana*) in Italy. Phytopathol. Mediterr. 62, 47–63. doi: 10.36253/phyto-14057

[B28] Gramaje D. Eichmeier A. Spetik M. Carbone M. J. Bujanda R. Vallance J.. (2022). Exploring the temporal dynamics of the fungal microbiome in rootstocks, the lesser-known half of the grapevine crop. J. Fungi 8, 421. doi: 10.3390/jof8050421 PMC9144578 35628677

[B29] Gramaje D. Úrbez-Torres J. R. Sosnowski M. R. (2018). Managing grapevine trunk diseases with respect to etiology and epidemiology: Current strategies and future prospects. Plant Dis. 102, 12–39. doi: 10.1094/PDIS-04-17-0512-FE 30673457

[B30] Guarnaccia V. Kraus C. Markakis E. Alves A. Armengol J. Eichmeier A.. (2022). Fungal trunk diseases of fruit trees in Europe: pathogens, spread and future directions. Phytopathol. Mediterr. 61, 563–599. doi: 10.36253/phyto-14167

[B31] Gusella G. Aiello D. Polizzi G. (2020). First report of leaf and twig blight of Indian hawthorn (*Rhaphiolepis indica*) caused by *Neofusicoccum parvum* in Italy. J. Plant Pathol. 102, 275–275. doi: 10.1007/s42161-019-00412-5

[B32] Haidar R. Yacoub A. Pinard A. Roudet J. Fermaud M. Rey P. (2020). Synergistic effects of water deficit and wood-inhabiting bacteria on pathogenicity of the grapevine trunk pathogen. Neofusicoccum parvum. Phytopathol. Mediterr. 59, 473–4845. doi: 10.14601/Phyto-12370

[B33] Hernández M. Kc A. N. (2024). Determining the timing of spore release by *Botryosphaeriaceae* species in Oregon Vineyards. Plant Dis. 108, 1033–1040. doi: 10.1094/PDIS-07-23-1359-RE 37923978

[B34] Inderbitzin P. Harkness J. Turgeon B. G. Berbee M. L. (2005). Lateral transfer of mating system in Stemphylium. Proc. Natl. Acad. Sci. United States America 102, 11390–113955. doi: 10.1073/pnas.0501918102 PMC1183548 16055562

[B35] Landis J. R. Koch G. G. (1977). The measurement of observer agreement for categorical data. Biometrics 33, 159–174. doi: 10.2307/2529310 843571

[B36] Linaldeddu B. T. Deidda A. Scanu B. Franceschini A. Alves A. Abdollahzadeh J.. (2016). Phylogeny, morphology and pathogenicity of *Botryosphaeriaceae*, *Diatrypaceae* and *Gnomoniaceae* associated with branch diseases of hazelnut in Sardinia (Italy). Eur. J. Plant Pathol. 146, 259–279. doi: 10.1007/s10658-016-0912-z

[B37] Linaldeddu B. T. Rossetto G. Maddau L. Vatrano T. Bregant C. (2023). Diversity and pathogenicity of. Botryosphaeriaceae Phytophthora species associated emerging olive Dis. Italy. Agriculture-Basel 13, 15755. doi: 10.3390/agriculture13081575

[B38] Luo Y. Gu S. Felts D. Puckett R. D. Morgan D. P. Michailides T. J. (2017). Development of qPCR systems to quantify shoot infections by canker-causing pathogens in stone fruits and nut crops. J. Appl. Microbiol. 122, 416–428. doi: 10.1111/jam.2017.122.issue-2 27862716

[B39] Luo Y. Lichtemberg P. S. F. Niederholzer F. J. A. Lightle D. M. Felts D. G. Michailides T. J. (2019). Understanding the process of latent infection of canker-causing pathogens in stone fruit and nut crops in California. Plant Dis. 103, 2374–2384. doi: 10.1094/PDIS-11-18-1963-RE 31306090

[B40] Luo Y. Niederholzer F. Camiletti B. X. Michailides T. J. (2024). Survey on latent infection of canker-causing pathogens in budwood and young trees from almond and prune nurseries in California. Plant Dis. 108, 550–5575. doi: 10.1094/PDIS-07-23-1449-SR 37807086

[B41] Márquez N. Giachero M. L. Declerck S. Ducasse D. A. (2021). *Macrophomina phaseolina*: General characteristics of pathogenicity and methods of control. Front. Plant Sci. 12. doi: 10.3389/fpls.2021.634397 PMC8100579 33968098

[B42] Marsberg A. Kemler M. Jami F. Nagel J. H. Postma-Smidt A. Naidoo S.. (2017). Botryosphaeria dothidea: a latent pathogen of global importance to woody plant health. Mol. Plant Pathol. 18, 477–4885. doi: 10.1111/mpp.12495 27682468 PMC6638292

[B43] Mesguida O. Haidar R. Yacoub A. Dreux-Zigha A. Berthon J. Y. Guyoneaud R.. (2023). Microbial biological control of fungi associated with grapevine trunk diseases: A review of strain diversity, modes of action, and advantages and limits of current strategies. J. Fungi 9, 6385. doi: 10.3390/jof9060638 PMC10299619 37367574

[B44] Moral J. Morgan D. Trapero A. Michailides T. J. (2019). Ecology and epidemiology of diseases of nut crops and olives caused by Botryosphaeriaceae fungi in California and Spain. Plant Dis. 103, 1809–1827. doi: 10.1094/PDIS-03-19-0622-FE 31232653

[B45] Ni H.-F. Yang H.-R. Chen R.-S. Hung T.-H. Liou R.-F. (2012). A nested multiplex PCR for species-specific identification and detection of Botryosphaeriaceae species on mango. Eur. J. Plant Pathol. 133, 819–828. doi: 10.1007/s10658-012-0003-8

[B46] Olmo D. Armengol J. León M. Gramaje D. (2016). Characterization and pathogenicity of Botryosphaeriaceae species isolated from almond trees on the island of Mallorca (Spain). Plant Dis. 100, 2483–24915. doi: 10.1094/PDIS-05-16-0676-RE 30686161

[B47] Pintos C. Redondo V. Costas D. Aguin O. Mansilla P. (2018). Fungi associated with grapevine trunk diseases in nursery-produced Vitis vinifera plants. Phytopathol. Mediterr. 57, 407–4245. Available at: https://www.jstor.org/stable/26675704.

[B48] Polizzi G. Di Pietro C. Gusella G. Ismail A. M. Aiello D. (2023). First report of seedling stem blight of mango caused by. Neofusicoccum parvum Italy. Plant Dis. 107, 16305. doi: 10.1094/PDIS-07-22-1652-PDN

[B49] Pouzoulet J. Rolshausen P. E. Schiavon M. Bol S. Travadon R. Lawrence D. P.. (2017). A Method to detect and quantify *Eutypa lata* and *Diplodia seriata*-complex DNA in grapevine pruning wounds. Plant Dis. 101, 1470–1480. doi: 10.1094/PDIS-03-17-0362-RE 30678588

[B50] Ramírez-Gil J. G. Osorio J. G. M. (2021). Source of inoculum of pathogens, the origin of disorders and diseases management in avocado nurseries. Australas. Plant Pathol. 50, 457–468. doi: 10.1007/s13313-021-00796-y

[B51] Reveglia P. Billones-Baaijens R. Savocchia S. (2022). Phytotoxic metabolites produced by fungi involved in grapevine trunk diseases: Progress, challenges, and opportunities. Plants-Basel 11, 3382. doi: 10.3390/plants11233382 36501420 PMC9736528

[B52] Ridgway H. J. Amponsah N. T. Brown D. S. Baskarathevan J. Jones E. E. Jaspers M. V. (2011). Detection of botryosphaeriaceous species in environmental samples using a multi-species primer pair. Plant Patholology 60, 1118–1127. doi: 10.1111/j.1365-3059.2011.02474.x

[B53] Romero-Cuadrado L. López-Herrera C. J. Aguado A. Capote N. (2023). Duplex real-time PCR assays for the simultaneous detection and quantification of Botryosphaeriaceae species causing canker diseases in woody crops. Plants 12, 2205. doi: 10.3390/plants12112205 37299184 PMC10255876

[B54] Slippers B. Wingfield M. (2007). Botryosphaeriaceae as endophytes and latent pathogens of woody plants: diversity, ecology and impact. Fungal Biol. Rev. 21, 90–106. doi: 10.1016/j.fbr.2007.06.002

[B55] Smahi H. Belhoucine-Guezouli L. Berraf-Tebbal A. Chouih S. Arkam M. Franceschini A.. (2017). Molecular characterization and pathogenicity of Diplodia corticola and other *Botryosphaeriaceae* species associated with canker and dieback of *Quercus suber* in Algeria. Mycosphere 8, 1261–1272. doi: 10.5943/mycosphere/8/2/10

[B56] Sosso J. Zakeel M. C. M. Akinsanmi O. A. (2021). Culturable fungal endophytes in Australian macadamia nursery plants. Australas. Plant Pathol. 50, 739–746. doi: 10.1007/s13313-021-00824-x

[B57] Tennakoon K. M. S. Ridgway H. J. Jaspers M. V. Jones E. E. (2018). *Botryosphaeriaceae* species associated with blueberry dieback and sources of primary inoculum in propagation nurseries in New Zealand. Eur. J. Plant Pathol. 150, 363–374. doi: 10.1007/s10658-017-1283-9

[B58] Thiessen L. D. Neill T. M. Mahaffee W. F. (2017). Timing fungicide application intervals based on airborne. Erysiphe necator concentrations. Plant Dis. 101, 1246–12525. doi: 10.1094/PDIS-12-16-1727-RE 30682951

[B59] Úrbez-Torres J. R. (2011). The status of *Botryosphaeriaceae* species infecting grapevines. Phytopathol. Mediterr. 50, S5–S45. Available at: http://www.jstor.org/stable/26458709.

[B60] Úrbez-Torres J. R. Battany M. Bettiga L. J. Gispert C. McGourty G. Roncoroni J.. (2010). Botryosphaeriaceae species spore-trapping studies in California vineyards. Plant Dis. 94, 717–724. doi: 10.1094/PDIS-94-6-0717 30754317

[B61] Úrbez-Torres J. R. Castro-Medina F. Mohali S. R. Gubler W. D. (2016). Botryosphaeriaceae species associated with cankers and dieback symptoms of *Acacia mangium* and *Pinus caribaea* var. *hondurensis* in Venezuela. Plant Dis. 100, 2455–24645. doi: 10.1094/PDIS-05-16-0612-RE 30686180

[B62] Úrbez-Torres J. R. Leavitt G. M. Voegel T. M. Gubler W. D. (2006). Identification and distribution of Botryosphaeria spp. associated with grapevine cankers in California. Plant Dis. 90, 1490–15035. doi: 10.1094/PD-90-1490 30780967

[B63] Valencia A. L. Gil P. M. Latorre B. A. Rosales I. M. (2019). Characterization and pathogenicity of Botryosphaeriaceae species obtained from avocado trees with branch canker and dieback and from avocado fruit with stem end rot in Chile. Plant Dis. 103, 996–1005. doi: 10.1094/PDIS-07-18-1131-RE 30840843

[B64] Velásquez A. C. Castroverde C. D. M. He S. Y. (2018). Plant-Pathogen warfare under changing climate conditions. Curr. Biol. 28, R619–R634. doi: 10.1016/j.cub.2018.03.054 29787730 PMC5967643

[B65] Venbrux M. Crauwels S. Rediers H. (2023). Current and emerging trends in techniques for plant pathogen detection. Front. Plant Sci. 14. doi: 10.3389/fpls.2023.1120968 PMC10200959 37223788

[B66] Vettraino A. M. Li H. M. Eschen R. Morales-Rodríguez C. Vannini A. (2017). The sentinel tree nursery as an early warning system for pathway risk assessment: Fungal pathogens associated with Chinese woody plants commonly shipped to Europe. PloS One 12, e0188800. doi: 10.1371/journal.pone.0188800 29186190 PMC5706704

[B67] White T. J. Bruns T. D. B. Lee S. Taylor W. J. (1990). “Amplification and direct sequencing of fungal ribosomal RNA genes for phylogenetics,” in PCR protocols: A guide to methods and applications, eds. Innis M. A. Gelfand D. H. (London: Academic Press), 315–322.

[B68] Zhang Q. Zhang Y. Shi H. Huo Y. (2023). Botryosphaeria dothidea causing leaf blight of Photinia serrulata in China. Crop Prot. 174, 106412. doi: 10.1016/j.cropro.2023.106412

