# Supplementary material for: Triplex real-time qPCR for the simultaneous detection of Botryosphaeriaceae species in woody crops and environmental samples
Source: Front Plant Sci. 2024 Oct 11;15:1435462. doi: 10.3389/fpls.2024.1435462 (PMC11502354; doi:10.3389/fpls.2024.1435462)
Supplement: Supplementary file 1 [file Table1.docx]

**Table S1.** Fungal isolates used in this study for the validation of the triplex qPCR protocol.

| **Species** | **Isolate ID** | **Collection^a^** | **Host** | **Triplex qPCR^b^** | | |
| --- | --- | --- | --- | --- | --- | --- |
|  |  |  |  | ***Bd*** | ***Np*** | **BOT** |
| *Botryosphaeria dothidea* | Bd ALM1 | TOR | *Prunus dulcis* | + | - | + |
|  | Bd ALM2 | TOR | *Prunus dulcis* | + | - | + |
|  | Bd ALM3 | TOR | *Prunus dulcis* | + | - | + |
|  | Bd ALM4 | TOR | *Prunus dulcis* | + | - | + |
|  | Bd ALM6 | TOR | *Prunus dulcis* | + | - | + |
|  | Bd ALM7 | TOR | *Prunus dulcis* | + | - | + |
|  | Bd ALM8 | TOR | *Prunus dulcis* | + | - | + |
|  | Bd ALM9 | TOR | *Prunus dulcis* | + | - | + |
|  | Bd ALM10 | TOR | *Prunus dulcis* | + | - | + |
|  | Bd ALM11 | TOR | *Prunus dulcis* | + | - | + |
|  | Bd ALM12 | TOR | *Prunus dulcis* | + | - | + |
|  | Bd ALM13 | TOR | *Prunus dulcis* | + | - | + |
|  | Bd ALM14 | TOR | *Prunus dulcis* | + | - | + |
|  | Bd ALM15 | TOR | *Prunus dulcis* | + | - | + |
|  | Bd ALM16 | TOR | *Prunus dulcis* | + | - | + |
|  | Bd ALM17 | TOR | *Prunus dulcis* | + | - | + |
|  | ALM TOR1 | TOR | *Prunus dulcis* | + | - | + |
|  | FC10B | TOR | *Prunus dulcis* | + | - | + |
|  | FC10G | TOR | *Prunus dulcis* | + | - | + |
|  | FC10C | TOR | *Prunus dulcis* | + | - | + |
|  | FC10I | TOR | *Prunus dulcis* | + | - | + |
|  | Bo.13.2 | ETSIA | *Vaccinium corymbosum* | + | - | + |
|  | Bd1141 | - | *Vitis vinifera* | + | - | + |
|  | Bd1143 | - | *Vitis vinifera* | + | - | + |
| *Neofusicoccum australe* | Bo.8 | ETSIA | *Vaccinium corymbosum* | - | - | + |
| *Neofusicoccum luteum* | NF 146 | IAS | *Persea americana* | - | - | + |
| *Neofusicoccum mediterraneum* | Nm ALM3 | TOR | *Prunus dulcis* | - | - | + |
|  | CJL 593 | IRTA | *Pistacia vera* | - | - | + |
|  | CH_LT_044 | ITACYL | *Vitis vinifera* | - | - | + |
| *Neofusicoccum parvum* | Np ALM1 | TOR | *Prunus dulcis* | - | + | + |
|  | Np ALM2 | TOR | *Prunus dulcis* | - | + | + |
|  | Np ALM5 | TOR | *Prunus dulcis* | - | + | + |
|  | NF 152 | IAS | *Persea americana* | - | + | + |
|  | NF 161 | IAS | *Persea americana* | - | + | + |
|  | Bo.2 | ETSIA | *Vaccinium corymbosum* | - | + | + |
|  | Bo.4.1 | ETSIA | *Vaccinium corymbosum* | - | + | + |
|  | Bo.4.2 | ETSIA | *Vaccinium corymbosum* | - | + | + |
|  | Bo.6.1 | ETSIA | *Vaccinium corymbosum* | - | + | + |
|  | Bo.7 | ETSIA | *Vaccinium corymbosum* | - | + | + |
|  | Bo.9 | ETSIA | *Vaccinium corymbosum* | - | + | + |
|  | Bo.10 | ETSIA | *Vaccinium corymbosum* | - | + | + |
|  | Bo.13.3 | ETSIA | *Vaccinium corymbosum* | - | + | + |
|  | Bo.14.2 | ETSIA | *Vaccinium corymbosum* | - | + | + |
|  | Bo.16 | ETSIA | *Vaccinium corymbosum* | - | + | + |
|  | Bo.17.1 | ETSIA | *Vaccinium corymbosum* | - | + | + |
|  | ARA21A | TOR | *Vaccinium corymbosum* | - | + | + |
|  | ARA21B | TOR | *Vaccinium corymbosum* | - | + | + |
|  | ARA22A | TOR | *Vaccinium corymbosum* | - | + | + |
|  | ARA22B | TOR | *Vaccinium corymbosum* | - | + | + |
|  | CH_LT_039 | ITACYL | *Vitis vinifera* | - | + | + |
|  | CH_LT_057a | ITACYL | *Vitis vinifera* | - | + | + |
| *Neofusicoccum stellenboschiana* | 130.2 | ICIA | *Persea americana* | - | - | + |
|  | 186.3 | ICIA | *Persea americana* | - | - | + |
|  | 192.1.1 | ICIA | *Persea americana* | - | - | + |
| *Diplodia cortícola* | CJL 165 | IRTA | *Quercus suber* | - | - | + |
|  | CJL 166 | IRTA | *Quercus suber* | - | - | + |
| *Diplodia coryli* | CH_LT_053 | ITACYL | *Vitis vinifera* | - | - | + |
| *Diplodia cupresii* | GIHF 321 | UPV | *Vitis vinifera* | - | - | + |
| *Diplodia mutila* | CJL 456 | IRTA | *Fraxinus excelsior* | - | - | + |
|  | CH_LT_036 | ITACYL | *Vitis vinifera* | - | - | + |
| *Diplodia sapinea* | CH_LT_058 | ITACYL | *Pinus* | - | - | + |
| *Diplodia seriata* | Ds ALM1 | TOR | *Prunus dulcis* | - | - | + |
|  | L4-1 | TOR | *Prunus dulcis* | - | - | + |
|  | S1-1 | TOR | *Prunus dulcis* | - | - | + |
|  | S2-1 | TOR | *Prunus dulcis* | - | - | + |
|  | CJL 398 | IRTA | *Vitis vinifera* | - | - | + |
| *Dothiorella fraxini* | GIHF 132 | UPV | *Fraxinus angustifolia* | - | - | + |
| *Dothiorella iberica* | CJL 218 | IRTA | *Quercus ilex* | - | - | + |
|  | CJL 220 | IRTA | *Quercus ilex* | - | - | + |
| *Dothiorella sarmentorum* | CH_LT_037 | ITACYL | *Vitis vinifera* | - | - | + |
| *Dothiorella viticola* | CJL 570 | IRTA | *Vitis vinifera* | - | - | + |
|  | CJL 572 | IRTA | *Vitis vinifera* | - | - | + |
|  | CH_LT_032 | ITACYL | *Vitis vinifera* | - | - | + |
|  | S1-2 | TOR | *Prunus dulcis* | - | - | + |
| *Lasiodiplodia theobromae* | L.2 | TOR | *Vaccinium corymbosum* | - | - | + |
|  | ARA 2 | TOR | *Vaccinium corymbosum* | - | - | + |
|  | ARA 3 | TOR | *Vaccinium corymbosum* | - | - | + |
|  | ARA 4 | TOR | *Vaccinium corymbosum* | - | - | + |
|  | ARA 6 | TOR | *Vaccinium corymbosum* | - | - | + |
|  | ARA 8 | TOR | *Vaccinium corymbosum* | - | - | + |
|  | ARA 9 | TOR | *Vaccinium corymbosum* | - | - | + |
|  | ARA 10 | TOR | *Vaccinium corymbosum* | - | - | + |
|  | ARA 15 | TOR | *Vaccinium corymbosum* | - | - | + |
|  | ARA 17 | TOR | *Vaccinium corymbosum* | - | - | + |
|  | ARA 17B | TOR | *Vaccinium corymbosum* | - | - | + |
|  | GIHF 272 | UPV | *Vitis vinifera* | - | - | + |
| *Macrophomina phaseolina* | Mp ALM 1 | TOR | *Prunus dulcis* | - | - | + |
|  | Mp ALM 2 | TOR | *Prunus dulcis* | - | - | + |
|  | FC10P | TOR | *Prunus dulcis* | - | - | + |
|  | P2 RP7 | TOR | *Prunus dulcis* | - | - | + |
|  | Mp ARA11 | TOR | *Vaccinium corymbosum* | - | - | + |
|  | Mp ARA12 | TOR | *Vaccinium corymbosum* | - | - | + |
|  | TOR 872 | TOR | *Vaccinium corymbosum* | - | - | + |
|  | TOR 956 | TOR | *Vaccinium corymbosum* | - | - | + |
| *Phyllosticta fallopiae* | 29.3 | ICIA | *Persea americana* | - | - | - |
| *Cytospora acaciae* | Ca ALM1 | TOR | *Prunus dulcis* | - | - | - |
|  | Ca ALM2 | TOR | *Prunus dulcis* | - | - | - |
|  | Ca ALM3 | TOR | *Prunus dulcis* | - | - | - |
| *Botrytis cinerea* | Bc ARA1 | TOR | *Vaccinium corymbosum* | - | - | - |
|  | Bc ALM1 | TOR | *Prunus dulcis* | - | - | - |
| *Monilia fructicola* | Mf CIR1 | TOR | *Prunus salicina* | - | - | - |
| *Monilia laxa* | Ml CIR1 | TOR | *Prunus salicina* | - | - | - |
| *Diaporthe ambigua* | L4-2 | TOR | *Prunus dulcis* | - | - | - |
| *Diaporthe amygdali* | DAL-65 | UPV | *Prunus dulcis* | - | - | - |
| *Diaporthe foeniculina* | DAL-69 | UPV | *Prunus dulcis* | - | - | - |
| *Diaporthe phaseolorum* | DAL-222 | UPV | *Prunus dulcis* | - | - | - |
| *Collectotrichum accutatum* | 20240 | CECT | *-* | - | - | - |
| *Verticilium dahliae* | Vd ALM1 | TOR | *Prunus dulcis* | - | - | - |
| *Cylindrocladiella variabilis* | AL139 | TOR | *Prunus dulcis* | - | - | - |
| *Dactylonectria macrodidyma* | AL150 | TOR | *Prunus dulcis* | - | - | - |
| *Dactylonectria novozelandica* | AL84 | TOR | *Prunus dulcis* | - | - | - |
| *Dactylonectria torresensis* | AL3 | TOR | *Prunus dulcis* | - | - | - |
| *Ilyonectria liriodendri* | AL79 | TOR | *Prunus dulcis* | - | - | - |
| *Neonectria quercicola* | AL141 | TOR | *Prunus dulcis* | - | - | - |
| *Rhizoctonia solani* | Rs ALM4 | TOR | *Prunus dulcis* | - | - | - |
| *Epicoccum nigrum* | En ALM5 | TOR | *Prunus dulcis* | - | - | - |
| *Alternaria alternata* | Al ALM1 | TOR | *Prunus dulcis* | - | - | - |
| ^a^TOR: Andalusian Institute of Agricultural and Fisheries Research and Training (IFAPA) Las Torres; ETSIA: Escuela Técnica Superior de Ingeniería Agronómica de Sevilla; IAS-CSIC: Instituto de Agricultura Sostenible-Consejo Superior de Investigaciones Científicas; IRTA: Instituto de Investigación y Tecnología Agroalimentaria; ITACYL: Instituto Tecnológico Agrario de Castilla y León; ICIA: Instituto Canario de Investigaciones Agrarias; UPV: Universidad Politécnica de Valencia; CECT: Colección Española de Cultivos Tipo (https://www.uv.es/uvweb/coleccion-espanola-cultivos-tipo/es/coleccion-espanola-cultivos-tipo-1285872233521.html). | | | | | | |
|  |  |  |  |  |  |  |
|  |  |  |  |  |  |  |
|  |  |  |  |  |  |  |
|  |  |  |  |  |  |  |
| ^b^+: positive amplification; -: negative amplification; *Bd*: *Botryosphaeria dothidea*; *Np*: *Neofusicoccum parvum*; BOT*:* Family *Botryosphaeriaceae.* | | | | | | |
|  |  |  |  |  |  |  |

**Tabla S2.** Primers and TaqMan probes designed for the specific detection of *Botryosphaeriaceae* species by triplex qPCR.

| **Oligo name** | **Oligo type** | **Sequence 5'-3'^a^** | **Target organism** | **Target gene^b^** | **Length (bp)** | **TM**  **(ºC)** | **Amplicon**  **size** |
| --- | --- | --- | --- | --- | --- | --- | --- |
| Bd-F1 | Forward primer | CGCCGAATTTGCCTTATCA | *Botryosphaeria dothidea* | *tef* | 19 | 62 | 186 bp |
| Bd-R1 | Reverse primer | TTAGCATATGGTCGCATAGAC |  |  | 21 | 61 |  |
| Bd-P | Probe | **FAM**-TCACCAACG/ZEN/CTTCCAGCCACTCA-Iowa Black FQ |  |  | 23 | 70 |  |
|  |  |  |  |  |  |  |  |
| Np-F1 | Forward primer | GAAGTTCGAGAAGGTAAGAAAGT | *Neofusicoccum parvum* | *tef* | 23 | 62 | 74 bp |
| Np-R1 | Reverse primer | TGAGTGCGGGAACCC |  |  | 15 | 63 |  |
| Np-P | Probe | **HEX**-CTGCACGCG/ZEN/CTGGGTGCCAG-Iowa Black FQ |  |  | 20 | 72 |  |
|  |  |  |  |  |  |  |  |
| Bot-Bt-F1 | Forward primer | GTATGGCAATCTTCTGAACG | *Botryosphaeriaceae* family | *tub2* | 20 | 59 |  |
| Bot-Bt-R2 | Reverse primer | GAARAGCTGGCCRAAGG |  |  | 17 | 62 | 124 bp |
| Bot-Probe | Probe | **Cy5**-TCGAGCCCG/TAO/GCACSATGGAT-3IAbRQSp |  |  | 20 | 70 |  |
| ^a^R= A o G; S= G o C. | |  |  |  |  |  |  |
| ^b^*tef:* translation elongation factor 1 alpha; *tub*2: beta-tubulin. | | |  |  |  |  |  |

**Table S3.** Plant material analyzed using triplex qPCR for the applicability of the technique.

| **Crop** | **Sample name^a^** | **Origin** | **Tissue** | **Variety** | **Symptoms^b^** | **Triplex qPCR^c^** | | | **Isolation and sequencing^d^** | **Orchard Location** |
| --- | --- | --- | --- | --- | --- | --- | --- | --- | --- | --- |
|  |  |  |  |  |  | ***Bd*** | ***Np*** | **BOT** |  |  |
| Almond | ALMTOR1 | Production field | Trunk subcortical tissue | Marinada | S | **+** | **-** | **+** | *B. dothidea* | Alcalá del Río, Sevilla |
|  | ESol1 | Production field | Trunk subcortical tissue | Soleta | S | **+** | **-** | **+** | *B. dothidea* | Jerez de la Frontera, Cádiz |
|  | ESol2 | Production field | Trunk subcortical tissue | Soleta | S | **-** | **-** | **-** | NI | Jerez de la Frontera, Cádiz |
|  | ESol3 | Production field | Trunk subcortical tissue | Soleta | S | **-** | **-** | **-** | NI | Jerez de la Frontera, Cádiz |
|  | ESol4 | Production field | Trunk subcortical tissue | Soleta | S | **-** | **-** | **-** | NI | Jerez de la Frontera, Cádiz |
|  | ESol5 | Production field | Trunk subcortical tissue | Soleta | S | **-** | **+** | **+** | *N. parvum* | Jerez de la Frontera, Cádiz |
|  | ESol6 | Production field | Trunk subcortical tissue | Soleta | S | **-** | **-** | **-** | NI | Jerez de la Frontera, Cádiz |
|  | ESol7 | Production field | Trunk subcortical tissue | Soleta | S | **-** | **+** | **+** | *N. parvum* | Jerez de la Frontera, Cádiz |
|  | ESol8 | Production field | Trunk subcortical tissue | Soleta | A | **-** | **-** | **-** | NI | Jerez de la Frontera, Cádiz |
|  | ELau1 | Production field | Trunk subcortical tissue | Lauranne | S | **-** | **-** | **-** | NI | Jerez de la Frontera, Cádiz |
|  | ELau2 | Production field | Trunk subcortical tissue | Lauranne | S | **+** | **-** | **+** | *B. dothidea* | Jerez de la Frontera, Cádiz |
|  | ELau3 | Production field | Trunk subcortical tissue | Lauranne | S | **-** | **-** | **-** | NI | Jerez de la Frontera, Cádiz |
|  | ELau4 | Production field | Trunk subcortical tissue | Lauranne | S | **-** | **-** | **-** | NI | Jerez de la Frontera, Cádiz |
|  | ELau5 | Production field | Trunk subcortical tissue | Lauranne | A | **-** | **-** | **+** | NI | Jerez de la Frontera, Cádiz |
|  | ELau6 | Production field | Trunk subcortical tissue | Lauranne | S | **+** | **-** | **+** | *B. dothidea* | Jerez de la Frontera, Cádiz |
|  | MSol1 | Production field | Trunk subcortical tissue | Soleta | A | **-** | **-** | **-** | NI | La Rinconada, Sevilla |
|  | MSol2 | Production field | Trunk subcortical tissue | Soleta | A | **+** | **-** | **+** | *B. dothidea* | La Rinconada, Sevilla |
|  | MBel1 | Production field | Trunk subcortical tissue | Belona | A | **-** | **-** | **-** | NI | La Rinconada, Sevilla |
|  | MBel2 | Production field | Trunk subcortical tissue | Belona | A | **-** | **-** | **-** | NI | La Rinconada, Sevilla |
|  | MSol4-7 | Production field | Trunk subcortical tissue | Soleta | S | **+** | **-** | **+** | *B. dothidea* | La Rinconada, Sevilla |
|  | MSol4-10 | Production field | Trunk subcortical tissue | Soleta | S | **-** | **-** | **-** | NI | La Rinconada, Sevilla |
|  | MSol4-15 | Production field | Trunk subcortical tissue | Soleta | S | **-** | **-** | **-** | NI | La Rinconada, Sevilla |
|  | MSol4-18 | Production field | Trunk subcortical tissue | Soleta | S | **-** | **-** | **-** | NI | La Rinconada, Sevilla |
|  | MSol4-23 | Production field | Trunk subcortical tissue | Soleta | S | **-** | **-** | **-** | NI | La Rinconada, Sevilla |
|  | MBel2-6 | Production field | Trunk subcortical tissue | Belona | S | **+** | **-** | **+** | *B. dothidea* | La Rinconada, Sevilla |
|  | MBel3-13 | Production field | Trunk subcortical tissue | Belona | S | **+** | **-** | **+** | *B. dothidea* | La Rinconada, Sevilla |
|  | MBel3-20 | Production field | Trunk subcortical tissue | Belona | S | **+** | **-** | **+** | *B. dothidea* | La Rinconada, Sevilla |
|  | MBel4-7 | Production field | Trunk subcortical tissue | Belona | S | **-** | **-** | **-** | NI | La Rinconada, Sevilla |
|  | MBel4-11 | Production field | Trunk subcortical tissue | Belona | S | **+** | **-** | **+** | *B. dothidea* | La Rinconada, Sevilla |
|  | FC9_aerial | Production field | Trunk subcortical tissue | Lauranne | S | **-** | **-** | **-** | NI | Palma del Río, Córdoba |
|  | FC9_root | Production field | Root | Lauranne | S | **-** | **-** | **-** | NI | Palma del Río, Córdoba |
|  | FC10_aerial | Production field | Trunk subcortical tissue | Lauranne | S | **+** | **-** | **+** | *B. dothidea* | Palma del Río, Córdoba |
|  | FC10_root | Production field | Root | Lauranne | S | **-** | **-** | **+** | *M. phaseolina* | Palma del Río, Córdoba |
|  | FC11_aerial | Production field | Trunk subcortical tissue | Lauranne | S | **-** | **-** | **-** | NI | Palma del Río, Córdoba |
|  | FC11_root | Production field | Root | Lauranne | S | **-** | **-** | **-** | NI | Palma del Río, Córdoba |
|  | VSol 1 | Nursery | Grafting scion | Soleta | A | **-** | **-** | **-** | NI | Sevilla_June 2023 |
|  | VSol 2 | Nursery | Grafting scion | Soleta | A | **-** | **-** | **-** | NI | Sevilla_June 2023 |
|  | VSol 3 | Nursery | Grafting scion | Soleta | A | **-** | **-** | **-** | NI | Sevilla_June 2023 |
|  | VSol 4 | Nursery | Grafting scion | Soleta | A | **-** | **-** | **-** | NI | Sevilla_June 2023 |
|  | VSol 5 | Nursery | Grafting scion | Soleta | A | **-** | **-** | **-** | NI | Sevilla_June 2023 |
|  | VBel 1 | Nursery | Grafting scion | Belona | A | **-** | **-** | **-** | NI | Sevilla_June 2023 |
|  | VBel 2 | Nursery | Grafting scion | Belona | A | **-** | **-** | **-** | NI | Sevilla_June 2023 |
|  | VBel 3 | Nursery | Grafting scion | Belona | A | **-** | **-** | **-** | NI | Sevilla_June 2023 |
|  | VBel 4 | Nursery | Grafting scion | Belona | A | **-** | **-** | **-** | NI | Sevilla_June 2023 |
|  | VBel 5 | Nursery | Grafting scion | Belona | A | **-** | **-** | **-** | NI | Sevilla_June 2023 |
|  | VVai 1 | Nursery | Grafting scion | Vairo | A | **-** | **-** | **-** | NI | Sevilla_June 2023 |
|  | VVai 2 | Nursery | Grafting scion | Vairo | A | **-** | **-** | **-** | NI | Sevilla_June 2023 |
|  | VVai 3 | Nursery | Grafting scion | Vairo | A | **-** | **-** | **-** | NI | Sevilla_June 2023 |
|  | VVai 4 | Nursery | Grafting scion | Vairo | A | **-** | **-** | **-** | NI | Sevilla_June 2023 |
|  | VVai 5 | Nursery | Grafting scion | Vairo | A | **-** | **-** | **-** | NI | Sevilla_June 2023 |
|  | VMar 1 | Nursery | Grafting scion | Marcona | A | **-** | **-** | **-** | NI | Sevilla_June 2023 |
|  | VMar 2 | Nursery | Grafting scion | Marcona | A | **-** | **-** | **-** | NI | Sevilla_June 2023 |
|  | VMar 3 | Nursery | Grafting scion | Marcona | A | **-** | **-** | **-** | NI | Sevilla_June 2023 |
|  | VMar 4 | Nursery | Grafting scion | Marcona | A | **-** | **-** | **-** | NI | Sevilla_June 2023 |
|  | VMar 5 | Nursery | Grafting scion | Marcona | A | **-** | **-** | **-** | NI | Sevilla_June 2023 |
|  | VGua 1 | Nursery | Grafting scion | Guara | A | **-** | **-** | **-** | NI | Sevilla_June 2023 |
|  | VGua 2 | Nursery | Grafting scion | Guara | A | **-** | **-** | **-** | NI | Sevilla_June 2023 |
|  | VGua 3 | Nursery | Grafting scion | Guara | A | **-** | **-** | **-** | NI | Sevilla_June 2023 |
|  | VGua 4 | Nursery | Grafting scion | Guara | A | **-** | **-** | **-** | NI | Sevilla_June 2023 |
|  | VGua 5 | Nursery | Grafting scion | Guara | A | **-** | **-** | **-** | NI | Sevilla_June 2023 |
|  | VLau 1 | Nursery | Grafting scion | Lauranne | A | **-** | **-** | **-** | NI | Sevilla_June 2023 |
|  | VLau 2 | Nursery | Grafting scion | Lauranne | A | **-** | **-** | **-** | NI | Sevilla_June 2023 |
|  | VLau 3 | Nursery | Grafting scion | Lauranne | A | **-** | **-** | **-** | NI | Sevilla_June 2023 |
|  | VLau 4 | Nursery | Grafting scion | Lauranne | A | **-** | **-** | **-** | NI | Sevilla_June 2023 |
|  | VLau 5 | Nursery | Grafting scion | Lauranne | A | **-** | **-** | **-** | NI | Sevilla_June 2023 |
|  | VSol 1b | Nursery | Grafting scion | Soleta | A | **-** | **-** | **+** | *D. seriata / Do. viticola* | Sevilla_June 2023 |
|  | VSol 2b | Nursery | Grafting scion | Soleta | A | **-** | **-** | **+** | *D. seriata* | Sevilla_June 2023 |
|  | VSol 3b | Nursery | Grafting scion | Soleta | A | **-** | **-** | **-** | NI | Sevilla_June 2023 |
|  | VSol 4b | Nursery | Grafting scion | Soleta | A | **-** | **-** | **-** | NI | Sevilla_June 2023 |
|  | VSol 5b | Nursery | Grafting scion | Soleta | A | **-** | **-** | **-** | NI | Sevilla_June 2023 |
|  | VGua 1b | Nursery | Grafting scion | Guara | A | **-** | **-** | **-** | NI | Sevilla_June 2023 |
|  | VGua 2b | Nursery | Grafting scion | Guara | A | **-** | **-** | **-** | NI | Sevilla_June 2023 |
|  | VGua 3b | Nursery | Grafting scion | Guara | A | **-** | **-** | **-** | NI | Sevilla_June 2023 |
|  | VGua 4b | Nursery | Grafting scion | Guara | A | **-** | **-** | **-** | NI | Sevilla_June 2023 |
|  | VGua 5b | Nursery | Grafting scion | Guara | A | **-** | **-** | **-** | NI | Sevilla_June 2023 |
|  | VLau 1b | Nursery | Grafting scion | Lauranne | A | **-** | **-** | **-** | NI | Sevilla_June 2023 |
|  | VLau 2b | Nursery | Grafting scion | Lauranne | A | **-** | **-** | **-** | NI | Sevilla_June 2023 |
|  | VLau 3b | Nursery | Grafting scion | Lauranne | A | **-** | **-** | **-** | NI | Sevilla_June 2023 |
|  | VLau 4b | Nursery | Grafting scion | Lauranne | A | **-** | **-** | **+** | *D. seriata* | Sevilla_June 2023 |
|  | VLau 5b | Nursery | Grafting scion | Lauranne | A | **-** | **-** | **-** | NI | Sevilla_June 2023 |
|  | P1T | Nursery | Trunk subcortical tissue | GxN-15 | A | **-** | **-** | **-** | NI | Sevilla_June 2023 |
|  | P1RP | Nursery | Main root | GxN-15 | A | **-** | **-** | **-** | NI | Sevilla_June 2023 |
|  | P1Rs | Nursery | Secondary root | GxN-15 | A | **-** | **-** | **+** | NI | Sevilla_June 2023 |
|  | P2T | Nursery | Trunk subcortical tissue | GxN-15 | A | **-** | **-** | **-** | NI | Sevilla_June 2023 |
|  | P2RP | Nursery | Main root | GxN-15 | A | **-** | **-** | **+** | *M. phaseolina* | Sevilla_June 2023 |
|  | P2Rs | Nursery | Secondary root | GxN-15 | A | **-** | **-** | **-** | NI | Sevilla_June 2023 |
|  | P3T | Nursery | Trunk subcortical tissue | GxN-15 | A | **-** | **-** | **-** | NI | Sevilla_June 2023 |
|  | P3RP | Nursery | Main root | GxN-15 | A | **-** | **-** | **-** | *M. phaseolina* | Sevilla_June 2023 |
|  | P3Rs | Nursery | Secondary root | GxN-15 | A | **-** | **-** | **-** | NI | Sevilla_June 2023 |
|  | P4T | Nursery | Trunk subcortical tissue | GxN-15 | A | **-** | **-** | **-** | NI | Sevilla_June 2023 |
|  | P4RP | Nursery | Main root | GxN-15 | A | **-** | **-** | **-** | NI | Sevilla_June 2023 |
|  | P4Rs | Nursery | Secondary root | GxN-15 | A | **-** | **-** | **-** | NI | Sevilla_June 2023 |
|  | P5T | Nursery | Trunk subcortical tissue | GxN-15 | A | **-** | **-** | **-** | NI | Sevilla_June 2023 |
|  | P5RP | Nursery | Main root | GxN-15 | A | **-** | **-** | **-** | NI | Sevilla_June 2023 |
|  | P5Rs | Nursery | Secondary root | GxN-15 | A | **-** | **-** | **-** | NI | Sevilla_June 2023 |
|  | P6T | Nursery | Trunk subcortical tissue | GxN-15 | A | **-** | **-** | **-** | NI | Sevilla_June 2023 |
|  | P6RP | Nursery | Main root | GxN-15 | A | **-** | **-** | **-** | NI | Sevilla_June 2023 |
|  | P6Rs | Nursery | Secondary root | GxN-15 | A | **-** | **-** | **-** | NI | Sevilla_June 2023 |
|  | P7T | Nursery | Trunk subcortical tissue | GxN-15 | A | **-** | **-** | **-** | NI | Sevilla_June 2023 |
|  | P7RP | Nursery | Main root | GxN-15 | A | **-** | **-** | **+** | *M. phaseolina* | Sevilla_June 2023 |
|  | P7Rs | Nursery | Secondary root | GxN-15 | A | **-** | **-** | **+** | *M. phaseolina* | Sevilla_June 2023 |
|  | P8T | Nursery | Trunk subcortical tissue | GxN-15 | A | **-** | **-** | **-** | NI | Sevilla_June 2023 |
|  | P8RP | Nursery | Main root | GxN-15 | A | **-** | **-** | **-** | NI | Sevilla_June 2023 |
|  | P8Rs | Nursery | Secondary root | GxN-15 | A | **-** | **-** | **+** | NI | Sevilla_June 2023 |
|  | P9T | Nursery | Trunk subcortical tissue | GxN-15 | A | **-** | **-** | **-** | NI | Sevilla_June 2023 |
|  | P9RP | Nursery | Main root | GxN-15 | A | **-** | **-** | **-** | NI | Sevilla_June 2023 |
|  | P9Rs | Nursery | Secondary root | GxN-15 | A | **-** | **-** | **-** | NI | Sevilla_June 2023 |
|  | P10T | Nursery | Trunk subcortical tissue | GxN-15 | A | **-** | **-** | **-** | NI | Sevilla_June 2023 |
|  | P10RP | Nursery | Main root | GxN-15 | A | **-** | **-** | **-** | NI | Sevilla_June 2023 |
|  | P10Rs | Nursery | Secondary root | GxN-15 | A | **-** | **-** | **+** | *M. phaseolina* | Sevilla_June 2023 |
| Avocado | H1-H64 | Production field | Scion | Hass (n=64) | A | **-** | **-** | **-** | NI | Algarrobo, Málaga_Oct 2022 |
|  | R1-R56 | Production field | Scion | Reed (n=56) | A | **-** | **-** | **-** | NI | Algarrobo, Málaga_Oct 2022 |
|  | Avo1 | Production field | Scion | Hass | A | **-** | **-** | **+** | NI | Algarrobo, Málaga_Oct 2023 |
|  | Avo2 | Production field | Scion | Hass | A | **-** | **+** | **+** | NI | Algarrobo, Málaga_Oct 2023 |
|  | Avo3 | Production field | Scion | Hass | A | **-** | **-** | **+** | *L. theobromae* | Algarrobo, Málaga_Oct 2023 |
|  | Avo4 | Production field | Scion | Hass | A | **-** | **+** | **+** | NI | Algarrobo, Málaga_Oct 2023 |
|  | Avo5 | Production field | Scion | Hass | A | **-** | **+** | **+** | *N. parvum* | Algarrobo, Málaga_Oct 2023 |
|  | Avo6 | Production field | Scion | Hass | S | **-** | **+** | **+** | NI | Algarrobo, Málaga_Oct 2023 |
|  | Avo7 | Production field | Scion | Hass | S | **-** | **+** | **+** | NI | Algarrobo, Málaga_Oct 2023 |
|  | Avo8 | Production field | Scion | Hass | S | **-** | **+** | **+** | NI | Algarrobo, Málaga_Oct 2023 |
|  | Avo9 | Production field | Scion | Hass | S | **-** | **+** | **+** | NI | Algarrobo, Málaga_Oct 2023 |
|  | Avo10 | Production field | Scion | Hass | S | **-** | **+** | **+** | NI | Algarrobo, Málaga_Oct 2023 |
|  | Avo11 | Production field | Scion | Hass | S | **-** | **+** | **+** | NI | Algarrobo, Málaga_Oct 2023 |
|  | Avo12 | Production field | Scion | Hass | S | **-** | **+** | **+** | NI | Algarrobo, Málaga_Oct 2023 |
|  | Avo13 | Production field | Scion | Hass | S | **-** | **+** | **+** | *N. australe* | Algarrobo, Málaga_Oct 2023 |
|  | Avo14 | Production field | Scion | Hass | A | **-** | **-** | **-** | NI | Algarrobo, Málaga_Oct 2023 |
|  | Avo15 | Production field | Scion | Hass | A | **-** | **-** | **-** | NI | Algarrobo, Málaga_Oct 2023 |
|  | Avo16 | Production field | Scion | Hass | A | **-** | **-** | **-** | NI | Algarrobo, Málaga_Oct 2023 |
|  | Avo17 | Production field | Scion | Hass | A | **-** | **-** | **-** | NI | Algarrobo, Málaga_Oct 2023 |
|  | Avo18 | Production field | Scion | Hass | A | **-** | **-** | **-** | NI | Algarrobo, Málaga_Oct 2023 |
|  | Avo19 | Production field | Scion | Hass | A | **-** | **-** | **-** | NI | Algarrobo, Málaga_Oct 2023 |
|  | Avo20 | Production field | Scion | Hass | A | **-** | **-** | **-** | NI | Algarrobo, Málaga_Oct 2023 |
|  | Avo21 | Production field | Scion | Hass | A | **-** | **-** | **-** | NI | Algarrobo, Málaga_Oct 2023 |
|  | Avo22 | Production field | Scion | Hass | A | **-** | **-** | **-** | NI | Algarrobo, Málaga_Oct 2023 |
|  | Avo23 | Production field | Scion | Hass | A | **-** | **-** | **-** | NI | Algarrobo, Málaga_Oct 2023 |
|  | Avo24 | Production field | Scion | Hass | A | **-** | **-** | **-** | NI | Algarrobo, Málaga_Oct 2023 |
|  | Avo25 | Production field | Scion | Hass | A | **-** | **-** | **-** | NI | Algarrobo, Málaga_Oct 2023 |
|  | Avo26 | Production field | Scion | Hass | A | **-** | **-** | **-** | NI | Algarrobo, Málaga_Oct 2023 |
|  | Avo27 | Production field | Scion | Hass | A | **-** | **-** | **-** | NI | Algarrobo, Málaga_Oct 2023 |
|  | Avo28 | Production field | Scion | Hass | A | **-** | **-** | **-** | NI | Algarrobo, Málaga_Oct 2023 |
|  | Avo29 | Production field | Scion | Hass | A | **-** | **-** | **-** | NI | Algarrobo, Málaga_Oct 2023 |
|  | Avo30 | Production field | Scion | Hass | A | **-** | **-** | **-** | NI | Algarrobo, Málaga_Oct 2023 |
|  | Avo31 | Production field | Scion | Hass | A | **-** | **-** | **-** | NI | Algarrobo, Málaga_Oct 2023 |
|  | Avo32 | Production field | Scion | Hass | A | **-** | **-** | **-** | NI | Algarrobo, Málaga_Oct 2023 |
|  | Avo33 | Production field | Scion | Hass | A | **-** | **-** | **-** | NI | Algarrobo, Málaga_Oct 2023 |
|  | Avo34 | Production field | Scion | Hass | A | **-** | **-** | **-** | NI | Algarrobo, Málaga_Oct 2023 |
|  | Avo35 | Production field | Scion | Hass | A | **-** | **-** | **-** | NI | Algarrobo, Málaga_Oct 2023 |
|  | Avo36 | Production field | Scion | Hass | A | **-** | **-** | **-** | NI | Algarrobo, Málaga_Oct 2023 |
|  | Avo37 | Production field | Scion | Hass | A | **-** | **-** | **-** | NI | Algarrobo, Málaga_Oct 2023 |
|  | Avo38 | Production field | Scion | Hass | A | **-** | **-** | **-** | NI | Algarrobo, Málaga_Oct 2023 |
|  | Avo39 | Production field | Scion | Hass | A | **-** | **-** | **-** | NI | Algarrobo, Málaga_Oct 2023 |
|  | Avo40 | Production field | Scion | Hass | A | **-** | **-** | **-** | NI | Algarrobo, Málaga_Oct 2023 |
|  | Avo41 | Production field | Scion | Hass | A | **-** | **-** | **-** | NI | Algarrobo, Málaga_Oct 2023 |
|  | Avo42 | Production field | Scion | Hass | A | **-** | **-** | **-** | NI | Algarrobo, Málaga_Oct 2023 |
|  | Avo43 | Production field | Scion | Hass | A | **-** | **-** | **-** | NI | Algarrobo, Málaga_Oct 2023 |
|  | Avo44 | Production field | Scion | Hass | A | **-** | **-** | **-** | NI | Algarrobo, Málaga_Oct 2023 |
|  | Avo45 | Production field | Scion | Hass | A | **-** | **-** | **-** | NI | Algarrobo, Málaga_Oct 2023 |
|  | Avo46 | Production field | Scion | Hass | A | **-** | **-** | **-** | NI | Algarrobo, Málaga_Oct 2023 |
|  | Avo47 | Production field | Scion | Hass | A | **-** | **-** | **-** | NI | Algarrobo, Málaga_Oct 2023 |
|  | Avo48 | Production field | Scion | Hass | A | **-** | **-** | **-** | NI | Algarrobo, Málaga_Oct 2023 |
|  | Avo49 | Production field | Scion | Hass | S | **-** | **-** | **-** | NI | Algarrobo, Málaga_Oct 2023 |
|  | Avo50 | Production field | Scion | Hass | S | **-** | **-** | **-** | NI | Algarrobo, Málaga_Oct 2023 |
|  | Avo51 | Production field | Scion | Hass | S | **-** | **-** | **-** | NI | Algarrobo, Málaga_Oct 2023 |
|  | Avo52 | Production field | Scion | Hass | S | **-** | **-** | **-** | NI | Algarrobo, Málaga_Oct 2023 |
|  | Avo53 | Production field | Scion | Hass | S | **-** | **-** | **-** | NI | Algarrobo, Málaga_Oct 2023 |
|  | Avo54 | Production field | Scion | Hass | S | **-** | **-** | **-** | NI | Algarrobo, Málaga_Oct 2023 |
|  | Avo55 | Production field | Scion | Hass | S | **-** | **-** | **-** | NI | Algarrobo, Málaga_Oct 2023 |
|  | Avo56 | Production field | Scion | Hass | S | **-** | **-** | **-** | NI | Algarrobo, Málaga_Oct 2023 |
|  | Avo57 | Production field | Scion | Hass | S | **-** | **-** | **-** | NI | Algarrobo, Málaga_Oct 2023 |
|  | Avo58 | Production field | Scion | Hass | S | **-** | **-** | **-** | NI | Algarrobo, Málaga_Oct 2023 |
|  | Avo59 | Production field | Scion | Hass | S | **-** | **-** | **-** | NI | Algarrobo, Málaga_Oct 2023 |
|  | Avo60 | Production field | Scion | Hass | S | **-** | **-** | **-** | NI | Algarrobo, Málaga_Oct 2023 |
|  | Avo61 | Production field | Scion | Hass | S | **-** | **-** | **-** | NI | Algarrobo, Málaga_Oct 2023 |
|  | Avo62 | Production field | Scion | Hass | S | **-** | **-** | **-** | NI | Algarrobo, Málaga_Oct 2023 |
|  | Avo63 | Production field | Scion | Hass | S | **-** | **-** | **-** | NI | Algarrobo, Málaga_Oct 2023 |
|  | Avo64 | Production field | Scion | Hass | S | **-** | **-** | **-** | NI | Algarrobo, Málaga_Oct 2023 |
|  | Avo65 | Production field | Scion | Hass | A | **-** | **-** | **-** | NI | Algarrobo, Málaga_Oct 2023 |
|  | Avo66 | Production field | Scion | Hass | A | **-** | **-** | **-** | NI | Algarrobo, Málaga_Oct 2023 |
|  | Avo67 | Production field | Scion | Hass | A | **-** | **-** | **-** | NI | Algarrobo, Málaga_Oct 2023 |
|  | Avo68 | Production field | Scion | Hass | A | **-** | **-** | **-** | NI | Algarrobo, Málaga_Oct 2023 |
|  | Avo69 | Production field | Scion | Hass | A | **-** | **-** | **-** | NI | Algarrobo, Málaga_Oct 2023 |
|  | Avo70 | Production field | Scion | Hass | A | **-** | **-** | **-** | NI | Algarrobo, Málaga_Oct 2023 |
|  | Avo71 | Production field | Scion | Hass | A | **-** | **-** | **-** | NI | Algarrobo, Málaga_Oct 2023 |
|  | Avo72 | Production field | Scion | Hass | A | **-** | **-** | **-** | NI | Algarrobo, Málaga_Oct 2023 |
|  | Avo73 | Production field | Scion | Hass | A | **-** | **-** | **-** | NI | Algarrobo, Málaga_Oct 2023 |
|  | Avo74 | Production field | Scion | Hass | A | **-** | **-** | **-** | NI | Algarrobo, Málaga_Oct 2023 |
|  | Avo75 | Production field | Scion | Hass | S | **-** | **-** | **-** | NI | Algarrobo, Málaga_Oct 2023 |
|  | Avo76 | Production field | Scion | Hass | S | **-** | **-** | **-** | NI | Algarrobo, Málaga_Oct 2023 |
|  | Avo77 | Production field | Scion | Hass | S | **-** | **-** | **-** | NI | Algarrobo, Málaga_Oct 2023 |
|  | Avo78 | Production field | Scion | Hass | S | **-** | **-** | **-** | NI | Algarrobo, Málaga_Oct 2023 |
|  | Avo79 | Production field | Scion | Hass | S | **-** | **-** | **-** | NI | Algarrobo, Málaga_Oct 2023 |
|  | Avo80 | Production field | Scion | Hass | S | **-** | **-** | **-** | NI | Algarrobo, Málaga_Oct 2023 |
|  | Avo81 | Production field | Scion | Hass | S | **-** | **-** | **-** | NI | Algarrobo, Málaga_Oct 2023 |
|  | Avo82 | Production field | Scion | Hass | S | **-** | **-** | **-** | NI | Algarrobo, Málaga_Oct 2023 |
|  | Avo83 | Production field | Scion | Hass | S | **-** | **-** | **-** | NI | Algarrobo, Málaga_Oct 2023 |
|  | Avo84 | Production field | Scion | Hass | S | **-** | **-** | **-** | NI | Algarrobo, Málaga_Oct 2023 |
|  | Avo85 | Production field | Scion | Hass | S | **-** | **-** | **-** | NI | Algarrobo, Málaga_Oct 2023 |
|  | Avo86 | Production field | Scion | Hass | S | **-** | **-** | **-** | NI | Algarrobo, Málaga_Oct 2023 |
|  | Avo87 | Production field | Scion | Hass | S | **-** | **-** | **-** | NI | Algarrobo, Málaga_Oct 2023 |
|  | Avo88 | Production field | Scion | Hass | S | **-** | **-** | **-** | NI | Algarrobo, Málaga_Oct 2023 |
|  | Avo89 | Production field | Scion | Hass | S | **-** | **-** | **-** | NI | Algarrobo, Málaga_Oct 2023 |
|  | Avo90 | Production field | Scion | Hass | S | **-** | **-** | **-** | NI | Algarrobo, Málaga_Oct 2023 |
|  | Avo91 | Production field | Scion | Hass | S | **-** | **-** | **-** | NI | Algarrobo, Málaga_Oct 2023 |
|  | Avo92 | Production field | Scion | Hass | S | **-** | **-** | **-** | NI | Algarrobo, Málaga_Oct 2023 |
|  | Avo93 | Production field | Scion | Hass | S | **-** | **-** | **-** | NI | Algarrobo, Málaga_Oct 2023 |
|  | Avo94 | Production field | Scion | Hass | S | **-** | **-** | **-** | NI | Algarrobo, Málaga_Oct 2023 |
|  | Avo95 | Production field | Scion | Hass | S | **-** | **-** | **-** | NI | Algarrobo, Málaga_Oct 2023 |
|  | Avo96 | Production field | Scion | Hass | S | **-** | **-** | **-** | NI | Algarrobo, Málaga_Oct 2023 |
|  | Avo97 | Production field | Scion | Hass | S | **-** | **-** | **-** | NI | Algarrobo, Málaga_Oct 2023 |
|  | Avo98 | Production field | Scion | Hass | S | **-** | **-** | **-** | NI | Algarrobo, Málaga_Oct 2023 |
|  | Avo99 | Production field | Scion | Hass | S | **-** | **-** | **-** | NI | Algarrobo, Málaga_Oct 2023 |
|  | Avo100 | Production field | Scion | Hass | S | **-** | **-** | **-** | NI | Algarrobo, Málaga_Oct 2023 |
| Blueberry | Blu1T | Production field | Trunk subcortical tissue | Manila | S | **-** | **-** | **+** | *L. theobromae* | Moguer, Huelva |
|  | Blu1R | Production field | Root | Manila | S | **-** | **-** | **-** | NI | Moguer, Huelva |
|  | Blu7T | Production field | Trunk subcortical tissue | Manila | S | **-** | **-** | **-** | NI | Moguer, Huelva |
|  | Blu7R | Production field | Root | Manila | S | **-** | **-** | **-** | NI | Moguer, Huelva |
|  | Blu8T | Production field | Trunk subcortical tissue | Manila | S | **-** | **-** | **-** | NI | Moguer, Huelva |
|  | Blu8R | Production field | Root | Manila | S | **-** | **-** | **-** | NI | Moguer, Huelva |
|  | Blu2T | Production field | Trunk subcortical tissue | Ventura | S | **-** | **-** | **+** | *L. theobromae* | Moguer, Huelva |
|  | Blu2R | Production field | Root | Ventura | S | **-** | **-** | **-** | NI | Moguer, Huelva |
|  | Blu3T | Production field | Trunk subcortical tissue | Ventura | S | **-** | **-** | **+** | *L. theobromae* | Moguer, Huelva |
|  | Blu3R | Production field | Root | Ventura | S | **-** | **-** | **-** | NI | Moguer, Huelva |
|  | Blue5T | Production field | Trunk subcortical tissue | Ventura | S | **-** | **-** | **-** | NI | Moguer, Huelva |
|  | Blue5R | Production field | Root | Ventura | S | **-** | **-** | **-** | NI | Moguer, Huelva |
|  | Blue6T | Production field | Trunk subcortical tissue | Ventura | S | **-** | **-** | **-** | NI | Moguer, Huelva |
|  | Blue6T | Production field | Root | Ventura | S | **-** | **-** | **-** | NI | Moguer, Huelva |
|  | Blu9T | Production field | Trunk subcortical tissue | Cupla | S | **-** | **-** | **-** | NI | Moguer, Huelva |
|  | Blu9R | Production field | Root | Cupla | S | **-** | **-** | **-** | NI | Moguer, Huelva |
|  | Blu10T | Production field | Trunk subcortical tissue | Cupla | S | **-** | **-** | **-** | NI | Moguer, Huelva |
|  | Blu10R | Production field | Root | Cupla | S | **-** | **-** | **-** | NI | Moguer, Huelva |
|  | Blu11T | Production field | Trunk subcortical tissue | Cupla | S | **-** | **-** | **-** | NI | Moguer, Huelva |
|  | Blu11R | Production field | Root | Cupla | S | **-** | **-** | **-** | NI | Moguer, Huelva |
|  | Blu4T | Production field | Trunk subcortical tissue | MissAlice | S | **-** | **+** | **+** | *N. parvum* | Gibraleón, Huelva |
|  | Blu4R | Production field | Root | MissAlice | S | **-** | **-** | **-** | NI | Gibraleón, Huelva |
|  | Blu12T | Production field | Trunk subcortical tissue | Olympus | S | **-** | **-** | **-** | NI | Gibraleón, Huelva |
|  | Blu12R | Production field | Root | Olympus | S | **-** | **-** | **-** | NI | Gibraleón, Huelva |
|  | Blu15T | Production field | Trunk subcortical tissue | - | A | **-** | **-** | **-** | NI | Gibraleón, Huelva |
|  | Blu13T | Production field | Trunk subcortical tissue | Emerald | S | **-** | **-** | **-** | NI | Moguer, Huelva |
|  | Blu13R | Production field | Root | Emerald | S |  |  |  | NI | Moguer, Huelva |
|  | Blu14T | Production field | Trunk subcortical tissue | Emerald | S | **-** | **-** | **-** | NI | Moguer, Huelva |
|  | Blu14R | Production field | Root | Emerald | S |  |  |  | NI | Moguer, Huelva |
| Grapevine | TOR_01 | Production field | Shoot | Tempranillo | A | **-** | **-** | **-** | NI | Jerez de la Frontera, Cádiz |
|  | ARM_01 | Production field | Shoot | Tempranillo | A | **-** | **+** | **+** | NI | Jerez de la Frontera, Cádiz |
|  | LT_001 | Production field | Shoot | Tempranillo | S | **-** | **-** | **+** | NI | Jerez de la Frontera, Cádiz |
|  | TRU_01 | Production field | Shoot | Tempranillo | S | **-** | **+** | **+** | *N. parvum* | Trujillo, Cáceres |
|  | TT1 | Production field | Shoot | Tempranillo | S | **-** | **-** | **+** | *D. seriata* | Valladolid |
|  | TT2 | Production field | Shoot | Tempranillo | S | **-** | **-** | **+** | *D. seriata* | Valladolid |
|  | TT3 | Production field | Shoot | Tempranillo | S | **-** | **-** | **+** | *D. seriata* | Valladolid |
|  | TT4 | Production field | Shoot | Tempranillo | S | **-** | **-** | **+** | *D. seriata* | Valladolid |
|  | VD1 | Production field | Shoot | Verdejo | S | **-** | **-** | **+** | *D. seriata* | Valladolid |
|  | VD2 | Production field | Shoot | Verdejo | S | **-** | **-** | **+** | *D. seriata* | Valladolid |
|  | VD3 | Production field | Shoot | Verdejo | S | **-** | **-** | **+** | *D. seriata* | Valladolid |
|  | VD4 | Production field | Shoot | Verdejo | S | **-** | **-** | **+** | *D. seriata* | Valladolid |

^a^H1-H64 correspond to 64 'Hass' avocado samples collected in October 2022; R1-R56 correspond to 56 'Reed' avocado samples collected in October 2022.

^b^S: Symptomatic plant; A: Asymptomatic plant.

^c^+ positive detection; - no detection. Bd: Detection of *Botryosphaeria dothidea* with FAM fluorophore; Np: Detection of *Neofusicoccum parvum* with HEX fluorophore, BOT: Detection of *Botryosphaeriaceae* family with Cy5 fluorophore.

^d^ *B. dothidea*: *Botryosphaeria dothidea*; *N. parvum*: *Neofusicoccum parvum*; *M. phaseolina*: *Macrophomina phaseolina*; *D. seriata*: *Diplodia seriata*; *Do. viticola*: *Dothiorella viticola*; *L. theobromae*: *Lasiodiplodia theobromae.*

**Table S4.** Soil and rainwater samples analyzed by triplex qPCR for the applicability of the technique.

| **Crop** | **Sample name** | **Origin** | **Sampling date** | **Triplex qPCR^a^** | | | **Isolation and sequencing** |
| --- | --- | --- | --- | --- | --- | --- | --- |
|  |  |  |  | ***Bd*** | ***Np*** | **BOT** |  |
| Almond | S_B_FC9 | Soil |  | **-** | **-** | **+** | *Macrophomina phaseolina* |
|  | S_B_FC10 | Soil |  | **-** | **-** | **+** | *M. phaseolina* |
|  | S_B_FC11A | Soil |  | **-** | **-** | **+** | *M. phaseolina* |
|  | S_B_FC11B | Soil |  | **-** | **-** | **+** | *M. phaseolina* |
|  | S_B_FC11C | Soil |  | **-** | **-** | **+** | *M. phaseolina* |
|  | S_M_Sol 4-7 | Soil |  | **-** | **-** | **-** | NI |
|  | S_M_Bel 2-6 | Soil |  | **+** | **-** | **+** | NI |
|  | S_M_Bel 3-7 | Soil |  | **-** | **-** | **-** | NI |
|  | S_M_Bel 3-13 | Soil |  | **-** | **-** | **-** | NI |
|  | S_M_Bel 3-20 | Soil |  | **+** | **-** | **+** | NI |
| Avocado | S_LM_P10 | Soil |  | **-** | **-** | **+** | NI |
|  | S_LM_P16 | Soil |  | **-** | **-** | **+** | NI |
|  | S_LM_P20 | Soil |  | **-** | **-** | **+** | NI |
|  | S_LM_P22 | Soil |  | **-** | **-** | **+** | NI |
|  | S_LM_28.S9 | Soil |  | **-** | **+** | **+** | NI |
| Blueberry | S_EC_B2 | Soil |  | **-** | **-** | **+** | *M. phaseolina* |
|  | S_EC_A3 | Soil |  | **-** | **-** | **+** | *M. phaseolina* |
|  | S_EC_C4 | Soil |  | **-** | **-** | **-** | NI |
| Grapevine | S_LB_01 | Soil |  | **-** | **-** | **-** | NI |
|  | S_LB_02 | Soil |  | **-** | **-** | **-** | NI |
|  | S_LB_03 | Soil |  | **-** | **-** | **-** | NI |
|  | S_LT_01 | Soil |  | **-** | **-** | **-** | NI |
|  | S_LT_02 | Soil |  | **-** | **-** | **-** | NI |
|  | S_LT_03 | Soil |  | **+** | **+** | **+** | NI |
|  | S_RM_01 | Soil |  | **-** | **-** | **-** | NI |
|  | S_RM_02 | Soil |  | **-** | **-** | **-** | NI |
|  | S_RM_03 | Soil |  | **-** | **-** | **-** | NI |
| Almond | R_M_Sol 4-7 | Rainwater | November 2023 | **+** | **-** | **+** | NI |
|  |  | Rainwater | December 2023 | **-** | **-** | **+** | NI |
|  |  | Rainwater | January 2024 | **-** | **-** | **+** | NI |
|  |  | Rainwater | February 2024 | **-** | **-** | **-** | NI |
|  | R_M_Bel 2-6 | Rainwater | November 2023 | **-** | **-** | **+** | NI |
|  |  | Rainwater | December 2023 | **+** | **-** | **+** | NI |
|  |  | Rainwater | January 2024 | **-** | **-** | **+** | NI |
|  |  | Rainwater | February 2024 | **-** | **-** | **+** | NI |
|  | R_M_Bel 3-7 | Rainwater | November 2023 | **+** | **+** | **+** | NI |
|  |  | Rainwater | December 2023 | **-** | **-** | **+** | NI |
|  |  | Rainwater | January 2024 | **-** | **-** | **+** | NI |
|  |  | Rainwater | February 2024 | **-** | **-** | **+** | NI |
|  | R_M_Bel 3-13 | Rainwater | November 2023 | **+** | **-** | **+** | NI |
|  |  | Rainwater | December 2023 | **-** | **-** | **+** | NI |
|  |  | Rainwater | January 2024 | **-** | **-** | **+** | NI |
|  |  | Rainwater | February 2024 | **-** | **+** | **+** | NI |
|  | R_M_Bel 3-20 | Rainwater | November 2023 | **+** | **+** | **+** | NI |
|  |  | Rainwater | December 2023 | **-** | **-** | **+** | NI |
|  |  | Rainwater | January 2024 | **-** | **-** | **+** | NI |
|  |  | Rainwater | February 2024 | **-** | **-** | **+** | NI |

^a^+ positive detection; - no detection. NI: Non-identified; *Bd*: Detection of *Botryosphaeria dothidea* with FAM fluorophore; *Np*: Detection of *Neofusicoccum parvum* with HEX fluorophore, BOT: Detection of *Botryosphaeriaceae* family with Cy5 fluorophore.
